# Detoxification and stress response genes expressed in a western North American bumble bee, *Bombus huntii* (Hymenoptera: Apidae)

**DOI:** 10.1186/1471-2164-14-874

**Published:** 2013-12-12

**Authors:** Junhuan Xu, James P Strange, Dennis L Welker, Rosalind R James

**Affiliations:** 1 Department of Biology, Utah State University, 1410 N 800 E, North Logan, UT 84341, USA; 2 USDA-ARS, Pollinating Insects Research Unit, Department of Biology, Utah State University, Logan, UT 84322-5310, USA

**Keywords:** Bees, *Bombus huntii*, Bumble bees, Detoxification genes, Transcriptome

## Abstract

**Background:**

The Hunt bumble bee (*Bombus huntii* Greene, Hymenoptera: Apidae) is a holometabolous, social insect important as a pollinator in natural and agricultural ecosystems in western North America. Bumble bees spend a significant amount of time foraging on a wide variety of flowering plants, and this activity exposes them to both plant toxins and pesticides, posing a threat to individual and colony survival. Little is known about what detoxification pathways are active in bumble bees, how the expression of detoxification genes changes across life stages, or how the number of detoxification genes expressed in *B. huntii* compares to other insects.

**Results:**

We found *B. huntii* expressed at least 584 genes associated with detoxification and stress responses. The expression levels of some of these genes, such as those encoding the cytochrome P450s, glutathione S-transferases (GSTs) and glycosidases, vary among different life stages to a greater extent than do other genes. We also found that the number of P450s, GSTs and esterase genes expressed by *B. huntii* is similar to the number of these genes found in the genomes of other bees, namely *Bombus terrestris*, *Bombus impatiens*, *Apis mellifera* and *Megachile rotundata*, but many fewer than are found in the fly *Drosophila melanogaster.*

**Conclusions:**

*Bombus huntii* has transcripts for a large number of detoxification and stress related proteins, including oxidation and reduction enzymes, conjugation enzymes, hydrolytic enzymes, ABC transporters, cadherins, and heat shock proteins. The diversity of genes expressed within some detoxification pathways varies among the life stages and castes, and we typically identified more genes in the adult females than in larvae, pupae, or adult males, for most pathways. Meanwhile, we found the numbers of detoxification and stress genes expressed by *B. huntii* to be more similar to other bees than to the fruit fly. The low number of detoxification genes, first noted in the honey bee, appears to be a common phenomenon among bees, and perhaps results from their symbiotic relationship with plants. Many flowering plants benefit from pollinators, and thus offer these insects rewards (such as nectar) rather than defensive plant toxins.

## Background

In addition to pesticide resistance, detoxification and stress responses are important adaptations that allow insects to overcome the chemical defenses of the plants and animals they feed on. Genes associated with these responses have been identified in many insects, including the mosquitoes *Anopheles gambiae*[1] and *Aedes aegypti*[2], the fruit fly *Drosophila melanogaster*[3]*,* the honey bee *Apis mellifera*[4], and the red flour beetle *Tribolium castaneum*[5]. During pollination activities, bees are exposed to toxic substances in the environment, such as pesticides, phytochemicals, microbial toxins, pollutants and other xenobiotics [6-8], but genomic analyses of the honey bee, *A. mellifera*, found fewer detoxification genes than are present in *D. melanogaster* and *A. gambiae*[4,9]. To determine if this phenomenon is common among bees or unique to the honey bee, we evaluated the expression of detoxification and stress related genes in a common western North American bumble bee, *Bombus huntii* (the Hunt bumble bee). Like *A. mellifera*, *B. huntii* is a social insect with a diet based on pollen and nectar from a broad array of plants, so environmental exposures to xenobiotics should be similar. In addition, bumble bee susceptibility to pesticides has been found to be similar to that of *A. mellifera*[10].

**Figure 1:**
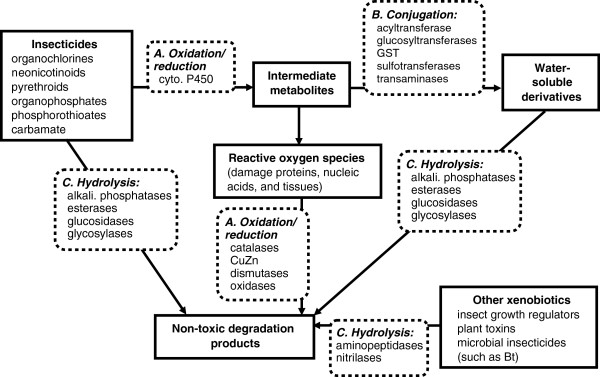
**Typical detoxification pathways known for insects.** Solid boxes indicate chemical compounds from the environment or as a result of metabolic processes. Dashed-line boxes indicate the three detoxification pathways that this paper focuses on.

Generally, stress responses and the detoxification of xenobiotics includes three major and interrelated pathways (Figure 1): oxidation-reduction, conjugation, and hydrolysis [11,12]. Oxidation-reduction enzymes include alcohol dehydrogenases, aldehyde dehydrogenases, cytochrome P450s, hydroxylases and peroxidases. Many cytochrome P450s are important enzymes for catalyzing oxidation-reduction reactions, and they are probably important for pesticide detoxification in *A. mellifera*[13]; however, reactive oxygen species (ROS), such as hydrogen peroxide, hydroxyl radicals, and superoxides, are typical by-products of these reactions. These ROS can be toxic in themselves, but are degraded by antioxidants, facilitated by other oxidation/reduction enzymes such as CuZn superoxide dismutases, catalases and peroxidases (Figure 1). In addition to the detoxification of insecticides and other xenobiotics, many of the oxidation/reduction enzymes are also involved in the normal physiological functioning of insects. For example, cytochrome P450s are also involved in the production of pheromones, ecdysteroids and juvenile hormones [14-16], and many of the enzymes that breakdown ROS are associated with breaking down by-products from metabolism.

The products of oxidation/reduction may be further degraded through conjugation. Conjugation is the process by which sugars, amino acids, or glutathione are transferred to a carrier compound for movement out of the cell. A host of conjugation enzymes catalyze these reactions [17] (Figure 1). Particularly important in insecticide detoxification are members of the Glutathione S-transferases enzyme (GST) superfamily which catalyze the conjugation of oxidized lipids and exogenous toxins, such as pyrethroid and organophosphate pesticides [18-20] (Figure 1). GSTs are also involved in other functions, such as intracellular transport and the biosynthesis of hormones [21].

Detoxification is also carried out via hydrolysis, which transfers a hydrogen atom from water to the substrate compound, and these reactions are facilitated by hydrolytic enzymes [12] (Figure 1). For example, carboxyl esterases catalyze the hydrolysis of esters into an acid and an alcohol and are important in the degradation of pyrethroids and organophosphate insecticides [22]. In addition to these three pathways, other pathways may be invoked, such as those that involve ATP-binding cassette transporters (ABC transporters) [11], cadherins, or heatshock proteins.

Like all bumble bees, the life stages and castes of *B. huntii* vary considerably in morphology, behavior and activity, and so could be expected to differ in their expression of detoxification and stress related genes. As with all Hymenoptera, bumble bees have a haplodiploid sex determination, where males are produced from unfertilized, haploid eggs and females from fertilized, diploid eggs [23]. Bumble bees live in annual, eusocial colonies founded by a single queen whose daughters form a female worker caste that provides foraging, brood care and nest maintenance [24]. A colony begins when a solitary overwintering queen emerges from hibernation, finds a suitable nesting site, provisions the nest with pollen and nectar, and commences laying eggs. The eggs hatch and larvae are fed nectar and pollen by the queen. In the fourth instar, the larvae begin to spin silk cocoons in which they pupate. The new adult bees emerge from their cells by chewing out of the cocoon, and these female workers then take over the responsibilities of brood care and foraging as the nest grows. Near the end of the summer, the next generation of reproductive adults is produced and the colony senesces. The new queens fly from the colony to mate with males and then diapause as solitary individuals in a suitable location for the winter, whereas workers, males and the colony’s original queen do not survive the winter.

Our goal was to construct a transcriptome of the detoxification and stress response genes expressed in *B. huntii*. However, due to the complex life cycle of bumble bees, it is possible that not all the relevant genes are expressed in a single caste or life stage. To identify a fuller spectrum of genes expressed in *B. huntii*, messenger RNA (mRNA) was extracted from eggs, early instar larvae, late instar larvae, pupae, adult workers, adult males, an egglaying queen, and a diapausing queen. The corresponding cDNA was sequenced using pyrosequencing, and genes associated with detoxification and stress response were identified. We also compared the number of cytochrome P450, GST and carboxylesterase genes found in *B. huntii* (acrosss all life stages) to those found in the fly *D. melanogaster*, and those found in other bees where annotated genomes are available, namely *B. terrestris*, *B. impatiens*, *A. mellifera*, and *M. rotundata,* to evaluate whether the low number of detoxification genes found in *A. mellifera*[4] is unique to that bee, or more common among bees in general.

## Results

### An overview of detoxification and stress response genes in *B. huntii*

We developed a cDNA database of 102,778 contigs and singletons using pyrosequencing of mRNA extracted from eggs, larvae, pupae, adult workers, adult males, an egglaying queen, and a diapausing queen of *B. huntii*. Gene expression data were examined two ways: the number of different genes detected within a class of detoxification genes (the gene diversity), and the number of transcripts of a particular gene or class of genes (the expression level). The total gene diversity within all the detoxification and stress response-related genes expressed in *B. huntii* was approximately 584 genes (Additional file 1: Table S1), after we removed sequences with high similarity to microorganisms and plants. Of the original detoxification and stress-related gene sequences we identified in the samples, 13.8% were bacterial, 12.4% were similar to known plant sequences and 3.6% were similar to known fungal sequences. These were all removed. Among the remaining genes, we identified genes associated with oxidation-reduction, conjugation, and hydrolytic enzymes, as well as some other detoxification and stress responses (Additional file 1: Table S1, Tables 1 and 2). The genes for enzymes in each of these four groups were expressed differently in the various life stages and castes, with some expressed in multiple stages and others expressed in only one or two (Tables 1 and 2).

**Table 1:** **Putative functions for the detoxification genes expressed in different life stages of *Bombus huntii*, based on the function assigned when the gene was first isolated**

**Enzymes**	**Putative functions**	**Taxon of identified gene**	**Reference**	***Bombus huntii *** **life stage/caste**^1^
**Oxidation and reduction enzymes**				
Aldehyde dehydrogenases	Acetaldehyde detoxification	*Drosophila melanogaster*	[25]	ii, iii, v, vi, vii
Alcohol dehydrogenases	Interconversion of alcohols and aldehydes or ketones with the reduction of NADH	*D. melanogaster*	[25]	all
Catalases	Catalyze H_2_O_2_ to H_2_O and O_2_	*Anopheles gambiae*	[26]	i, ii, iii, vi, vii, viii
Cytochrome P450s	Detoxify xenobiotics by transferring O_2_ to a substrate and producing H_2_O_2_, OH^–^, O_2_^-^	*D. melanogaster, A. gambiae, Tyria jacobaeae, Culex quinquefasciatus*	[1,16,27,28]	all
Dehalogenases	Remove halogen from haloacid compounds	*Sinorhizobium meliloti*	[29]	iii, iv, vi, vii
Hydroxylases	Degrades toxic organic compounds and pesticides by introducing –OH	*Chrysopogon zizanioides*	[30]	ii-vii
Oxidoreductases	Detoxify gramine	*Sitobion avenae*	[31]	all
Peroxidases	Catalyze H_2_O_2_ and organic hydroperoxides (OHPs)	*A. gambiae*	[32]	all
Superoxide dismutases (SOD)	Catalyze the dismutation of O_2_^–^ into O_2_ and H_2_O_2_	*A. mellifera*	[33,34]	all
Thioredoxins/glutaredoxins	Detoxification of ROS	*Saccharomyces cerevisiae*	[35]	i, ii, vi, vii, viii
**Conjugation enzymes**				
Acetyltransferases	Detoxify sulfadiazine and aromatic chemicals by transferring acetyl groups from acetyl-CoA to arylamines	*Legionella pneumophila, Homo sapiens*	[36,37]	i, ii, iii, v, vi, vii, viii
Acyltransferase	Catalyze carboxylic acid group, such as benzoic, isovaleric, or acetylsalicylic acids	*H. sapiens*	[38]	i, ii, iv, vi, vii, viii
CoA transferases	Acetate detoxification	*Aspergillus nidulans*	[39]	viii
Formyltransferases	Formaldehyde detoxification	*Burkholderia fungorum*	[40]	i, ii, iii, vi, vii, viii
Glutathione S-transferases (GST)	Detoxify endogenous compounds (peroxidised lipids) and exogenous toxins (pyrethroid and organophosphate) by catalyzing reduced glutathione	*Tenebrio molitor, Bombus ignites, Locusta migratoria manilensis*	[18,41]	all
Glycosyltransferases	Glycosyltransfer to OH–, NH_2_-, SH-, or COOH for detoxifying insecticides	*Bombyx mori*	[17,42]	all
Methyltransferases	Catalyze methylation reactions using S-adenosyl-L-methionine as a substrate for detoxification	*H. sapiens*	[43]	all
Phosphotransferases	Detoxify insecticides such as DDT	*Triatoma infestans*	[44]	i, ii, vi, vii, viii
Sulfotransferases	Detoxify insecticides such as DDT	*T. infestans*	[44]	iii, vii
Transaminases or aminotransferases	Detoxify 3-hydroxykynurenine and glyoxylate by catalyzing amino acid and α-keto acid	*Aedes aegypti, D. melanogaster*	[45]	ii-viii
**Hydrolytic enzymes**				
Acetylcholine and carboxyl esterases	Detoxify pyrethroids and organophosphates by hydrolysis of acyl group or ester bonds	*Lucilia cuprina, D. melanogaster*	[22]	all
Acid/Alkaline phosphatases	Detoxify endotoxin and plant toxin	*Danio rerio, Agriolimax agrestis*	[46,47]	ii, iii, vi, vii
Amidases	Detoxify toxic amides or esters, or organophosphorus or carbamate pesticides	*Acromyrmex octospinosus*	[48]	ii, iii, iv, vi, vii, viii
Aminopeptidases	Detoxify Bt Cry toxin, mycotoxin, organophosphonates, pyrethroid esters, microbial or botanical pesticides	*B. mori, Pseudoplusia includens, E. coli*	[42,48,49]	all
Cyclohydrolase	Hydrolysis of L-phenylalanine	*Drosophila spp.*	[50]	vi
Glycosidases	Hydrolyze sugar-containing compounds such as tannic acid and gallic acid	*Manduca sexta*	[48]	i, ii, iii, v, vi, vii, viii
Glycosylases	Detoxify cytotoxic and cytostatic substances such as 5-methyl group of a thymine residue, biodegrade saponins	*Phaeosphaeria nodorum, P. avenaria f. sp. triticea*	[51]	i, ii, vi, vii, viii
Nitrilases	Detoxify HCN	*Caenorhabditis elegans*	[52]	i-vii
Phosphodiesterase	Break phosphodiester bond for hydrolysis of organophosphate insecticides	*E. coli*	[53]	ii, iii, iv, vi, vii
Phosphohydrolases	Detoxify cypermethrin and bifenthrin	*Tribolium castaneum*	[54]	ii, iii-viii
**Others with possible stress related functions**				
ABC transporters	Export conjugated toxins out of the cell	*Drosophila spp.*	[11]	vii, viii
Cadherins	Resistance to Bt toxin (Cry1Ac)	*Helicoverpa armigera*	[55]	i, ii, vi, vii, viii
Heat shock proteins	Involved in folding and unfolding of proteins, detoxification of pesticides and heavy metals	*Chironomus tentans*	[56]	all
Isomerases	Detoxify organophosphates by structural rearrangement of isomers	*E. coli*	[16]	all
Lyases	Break various chemical bonds other than hydrolysis and oxidation for detoxification	*Zygaena transalpina*	[57]	all

i-egg, ii-early instar larvae, iii-late instar larvae, iv-pupae, v-adult male, vi-adult worker, vii-diapausing queen, viii-egglaying queen, all-all stages.

**Table 2:** **The number of detoxification genes identified, as expressed in different life stages of *Bombus huntii***

**Stages**	**Oxidation-reduction genes**	**Conjugation genes**	**Hydrolysis genes**	**Other detoxification genes**	**Total**
Egg	36	14	22	21	93
Early instars	49	20	33	23	125
Late instars	64	18	35	12	129
Pupa	31	9	13	7	60
Adult male	36	11	23	13	83
Adult worker	87	40	59	60	246
Diapausing queen	93	40	54	60	247
Egglaying queen	68	30	36	47	181
All stages	202	109	143	130	584

We found 202 genes associated with oxioreductases. Most of these were expressed across the majority of the life stages, but adult females had the greatest diversity of expressed gene types in this category (Table 2).

**Figure 2:**
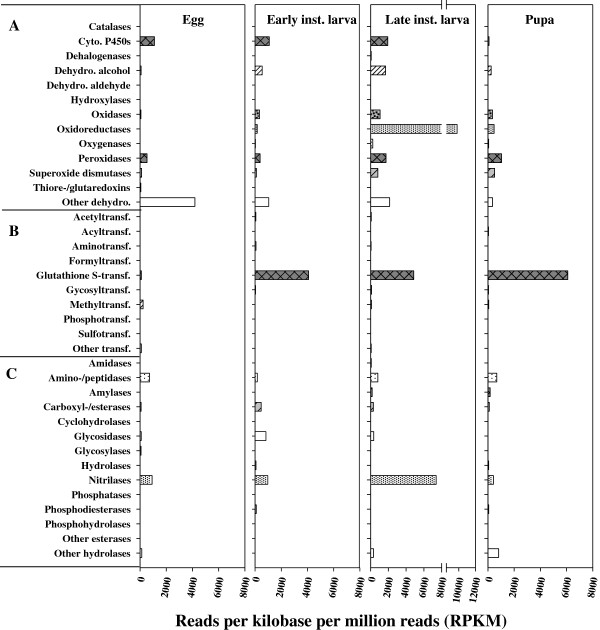
**The normalized expression levels of possible detoxification genes in *B. huntii *immatures, for genes in the (A) oxidation: reduction, (B) conjugation, and (C) hydrolysis groups.** All data were normalized to a million mapped reads per sample (1 RPKM = number of reads per million mapped reads of genes).

**Figure 3:**
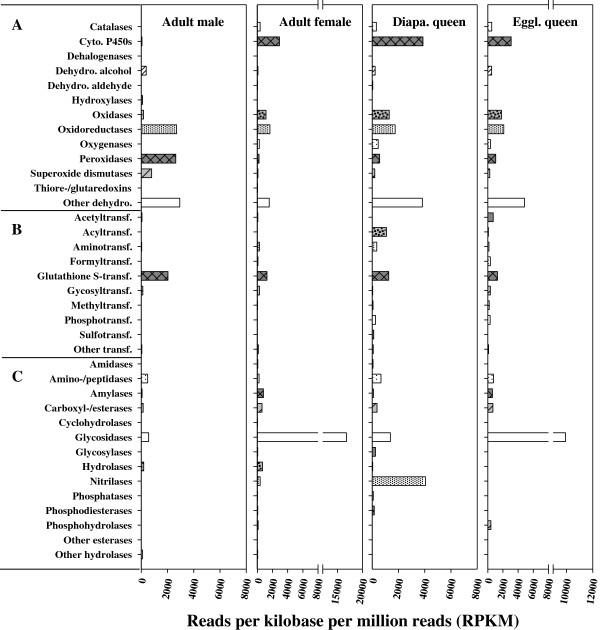
**The normalized expression levels of possible detoxification genes in *B. huntii *adults, for genes in the (A) oxidation: reduction, (B) conjugation, and (C) hydrolysis groups.** All data were normalized to a million mapped reads per sample (1 RPKM = number of reads per million mapped reads of genes).

Of the 109 conjugation-enzyme related genes we identified, GSTs, glycosyltransferases, methyltransferases and aminotransferases (transaminases) were expressed in all life stages. The genes coding for acetyltransferases, acyltransferases, CoA transferases, formyltransferases, and phosphotransferases were also expressed in different life stages, but abundantly in adult females (Figures 2 and 3). Genes encoding sulfotransferases were expressed only in the diapausing queen (Figures 2 and 3).

We detected a total of 143 genes for hydrolytic enzymes, across all castes and life stages (Tables 1 and 2), but the greatest diversity of expressed gene type was again found in the adult females (Table 2).

In addition to the genes in these three major groups, we found 130 other genes encoding proteins that potentially have a detoxification or stress response function, such as ABC transporters, cadherins, heat shock proteins, isomerases and lyases (Additional file 1: Table S1). Late instar larvae, pupae and adult males had the lowest diversity of these genes (Tables 1 and 2).

### Gene expression among the different life stages of *B. huntii*

The goal of this project was to survey detoxification and stress related genes in *B. huntii*. To do this, we used about 1000 eggs, 20–50 bees for the immature stages (see Methods), and one or two bees for the adult stages, and then pooled samples within each life stage for the 454 sequencing. Our sampling, which was not truly replicated within each life stage, provides some preliminary information on the expression levels among the different life stages and castes, but no statistical comparisons could be made. Among the immatures, the oxidoreductases were well expressed in the larvae, especially the late instar larvae (Figure 2). Within the conjugation enzyme group, the GSTs were highly expressed in the immatures, especially the pupae, but not the eggs (Figure 2). Genes associated with hydrolytic enzymes were also highly expressed in the immatures, especially the nitrilases, and again, particularly in the late instar larvae sample (Figure 2).

**Figure 4:**
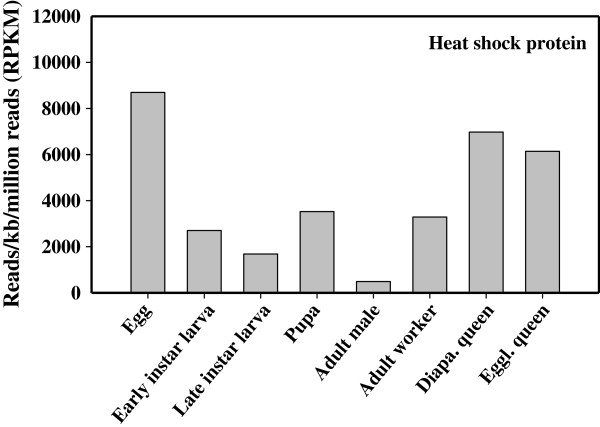
**Normalized expression levels of heat shock protein genes in *B. huntii *across all the life stages and castes.** All data were normalized to a million mapped reads per sample (1 RPKM = number of reads per million mapped reads of genes).

Genes associated with oxidation and reduction were well expressed in all the adult stages. The egglaying queen had quite high expression levels of these genes, although not for all the genes (Figure 3). As with the immatures, GSTs were the most well expressed genes in the conjugation enzyme group. In contrast to the high GST expression in the immatures, the glycosidases in adults were the most expressed of the hydrolytic enzymes, particularly in the adult workers and the egglaying queen (Figure 3). Heat shock protein genes were well expressed in the eggs, the diapausing queen and the egglaying queen, while expression levels in the males was fairly low (Figure 4).

### Comparison of detoxification genes in *B. huntii* with other insects

Gene diversity for detoxification and stress related genes in *B. huntii* was similar to, or lower than, that for the other bees (*B. terrestris*, *B. impatiens*, *A. mellifera* and *M. rotundata*), and generally a much lower gene diversity than *D. melanogaster* (Table 3). *Drosophila melanogaster* has many more P450s, acyltransferases, GSTs, and most of the hydrolases, than any of the bees. One exception is the nitrilases, for which we found a high diversity in *B. huntii*. The high nitrilase gene diversity does not occur in the other bees (Table 3). Our data was based on a transcriptome and may underrepresent the number of genes, relative to the number identified in the genomes of the other species included in this analysis.

**Table 3:** **Comparison of the number of detoxification genes identified in *Bombus huntii*, in other bees, and in *Drosophila melanogaster***

**Putative function**	***Bombus huntii***	***Bombus terrestris***	***Bombus impatiens***	***Apis mellifera***	***Megachile rotundata***	***Drosophila melanogaster***
**Oxidation and reduction enzymes**						
Aldehyde dehydrogenases	5	5	5	6	18	23
Catalases	2	1	2	6	5	3
Cytochrome P450	44	50	49	46	52	85
(CYP4)	(2)	(6)	(5)	(5)	(6)	(22)
(CYP6)	(18)	(22)	(22)	(28)	(19)	(22)
Glutaredoxins	2	3	7	4	3	7
Peroxidases	16	9	9	17	9	20
Superoxide dismutases	2	4	6	5	8	6
**Conjugation enzymes**						
Acyltransferases	13	28	30	32	8	55
Glutathione S-transferases	11	14	15	18	9	42
(Sigma-GSTs)	(3)	(0/?)	(0/?)	(4)	(1)	(1)
(Epsilon-GSTs)	(0)	(0/?)	(0/?)	(0)	(0)	(10)
Sulfotransferases	2	17	16	17	5	15
UDP-glucuronosyltransferases	2	6	8	2	2	7
**Hydrolytic enzymes**						
Alkaline phosphatases	1	3	4	3	2	16
Amidases	2	4	8	4	7	12
Aminopeptidases	12	19	26	24	27	41
Amylases	2	1	1	2	2	15
Carboxylesterases/Esterases	23	17	22	24	22	54
Glucosidases	8	7	8	18	5	29
Glucuronidases	1	1	1	1	2	4
Glycosylases	3	2	5	7	6	5
Nitrilases	25	1	2	3	2	2

Note: The numbers of *B. huntii* and *M. rotundata* genes are transcriptome data from 454 pyrosequencing, except for P450 of *M. rotundata* which is genomic data. The numbers of *B. terrestris*, *B. impatiens*, *A. mellifera*, and *D. melanogaster* genes are from genomic data from the NCBI GenBank database (http://www.ncbi.nlm.nih.gov/genome, accessed on September 25, 2013), and the numbers in brackets below P450 or GSTs are subgroups of P450 or GSTs.

We also compared some subgroups within the cytochrome P450s and GSTs. Within the cytochrome P450s, the CYP2 and mitochondrial P450 enzymes tend to have endogenous functions, while the CYP4 and CYP6 enzymes have detoxification functions. We found a similar number of CYP6 related genes among the six insects, however a greatly reduced number of CYP4 related genes in the bees, relative to *D. melanogaster* (Table 3). The GSTs are composed of several sub-groups, but the sigma-GSTs and epsilon-GSTs comprise the main sub-groups with detoxification functions. We found very few sigma-GSTs occurred in any of these insects; however, fewer epsilon-GSTs occurred in the bees than *D. melanogaster* (Table 3).

## Discussion

Female adults (workers and queens) had the greatest diversity and gene expression levels among the detoxification and stress related genes we identified in *B. huntii*. Female adults expressed high levels of putative detoxification genes, while adult males and pupae had relatively lower expression levels of these genes. The low diversity and expression levels in males may in part be associated with haploidy, as was demonstrated in the stingless bee *Melipona quadrifasciata*[58]. Behaviors associated with the different bumble bee castes may also affect gene expression. Male activities are all related to feeding themselves and mating; whereas, both workers and queens have more complex behaviors, often with high energy demands (e.g. foraging, brood care, and egglaying). Increases in foraging and feeding activity directly increase the risk of exposure to environmental pollutants; furthermore, increased energy demands have been shown to increase consumption rates and the formation of metabolic by-products in *A. mellifera*[59]. An actively laying queen may not forage after the first batch of brood matures, but she does have high metabolic demands for egglaying, policing the nest to keep workers from becoming egg layers, and incubating the brood. And, indeed, the diversity and expression of potential detoxification genes is relatively high in queens and workers.

Additionally, detoxification activity could be affected by variations in hormone levels and the complex morphological changes that occur in holometabolous insects. Changes of hormone levels during insect development are well quantified in *Drosophila*, with the steroid hormone ecdysone showing peaks during the transition from larva to pupa [60]. Metabolic activity also varies between life stages; most notably it declines during diapause, a dormant state during which respiration is very low. Thus, the fact that detoxification genes are more highly expressed in an egglaying than a diapausing queen is not entirely surprising. Non-diapausing adults are more active than pupae, but pupae undergo a major metamorphosis, so the relative expression of detoxification and stress related genes is more difficult to predict, but is more likely to be the result of metabolic processes and not a result of xenobiotic exposure.

Some detoxification mechanisms may be required more during certain developmental stages, and some genes that we classified as potential detoxification genes may serve non-detoxification functions. For example, glycosidases are more highly expressed in queens and workers than in other stages. Glucosidase catalyzes the cleavage of individual glucosyl residues from various glyco-conjugates, a process involved in the breakdown of sugar-containing compounds during the digestion of pollen and nectar. GSTs also break down a variety of compounds produced during metabolism and digestion, such as partially digested lipids, in addition to their involvement in the detoxification of environmental toxins, plant allelochemicals, and organochlorine and organophosphate insecticides [18-20]. GST genes are expressed at high levels in *B. huntii* adults, pupae, and larvae, but at low levels in eggs, which may be related to either differences in metabolism or exposure to environmental toxins. For example, late instar bee larvae accumulate high levels of lipids [61,62] and the high expression of GSTs in pupae may be related to the metabolism of these lipids during pupation. Similar results have been reported in other insects such as *A. aegypti, Lucilia cuprina,* and *Tenebrio molitor*, where the enzyme activities of GSTs were particularly high in the pupal stage [63]. In addition, some cytochrome P450s are involved in lipid metabolism, perhaps explaining why late instar larvae had higher expression levels of cytochrome P450s than early instar larvae.

Our gene analysis was based on a transcriptome, and as such, might underrepresent the number of detoxification genes found in the genome. However, we found similar numbers of genes to those found in the genomes of other bees. In addition, we used a large number of individuals for our transcriptome, and from across all the life stages of the bee. Thus, our transcriptome is probably a good library of the detoxification and stress related genes found in *B. huntii*. The fact that our bees were produced in culture on sugar syrup, rather than flower nectar, may have reduced the expression of some detoxification genes, such as has been found with honey bees [64]. However, we also fed our bees pollen collected by honey bees, and this likely provided a source of plant based phenolics and perhaps even traces of pesticides.

Due to the importance of detoxification capabilities during a bumble bee’s life, one might expect *B. huntii* to have a genome rich in detoxification genes; however, like other bees such as *A. mellifera*, *B. terrestris* and *B. impatiens*, we found *B. huntii* to have relatively few P450s, GSTs and carboxylesterases/esterases, as compared to *D. melanogaster*. For example, the number of P450 CYP4 genes is quite high in *D. melanogaster*, relative to what we found in *B. huntii. Bombus huntii* also has fewer epsilon-GSTs genes in comparison to *D. melanogaster*, although we found a slightly higher number of sigma-GSTs in *B. huntii* and *A. mellifera.* Epsilon-GSTs are known to be involved in DDT (dichlorodiphenyltrichloroethane) resistance and the detoxification of xenobiotics. The high number of epsilon-GSTs in *D. melanogaster* must reflect either a greater evolutionary diversification of these genes in the fruit fly, a loss of some genes in the bees, or a combination of both. In any case, this could arise if flies tend to have a greater exposure to environmental toxins than do the bees. Sigma-GSTs are associated with the detoxification of lipid peroxidation products, and provide protection from oxidants produced by the aerobic metabolism of honey, pollen and nectar in the bees [9]. Thus, the slightly greater number of sigma-GSTs in bees also appears to reflect their ecological niche.

We found here that the low number of detoxification genes found in the *A. mellifera* genome [4] is a phenomenon common to many bees. We hypothesize that this phenomenon may have arose as a result of the symbiotic relationship between bees and flowering plants. Flowering plants often produce rewards (such as nectar) to attract bees and other pollinators. Furthermore, at least some plants have lower levels of plant defensive compounds in the pollen and nectar [8], and adding plant alkaloids to the nectar reduces pollinator activity on those flowers [65]. Thus, the detoxification abilities of bees may be less than the flies (and likely other insects as well) due to a lower level of exposure to plant defensive compounds, compounds that plants produce to defend themselves against herbivores not pollinators.

## Conclusions

Using transcriptome analysis of all life stages, we found the Hunt bumble bee, *B. huntii*, to have the genetic potential to produce a large number of detoxification and stress related proteins, including oxidation and reduction enzymes, conjugation enzymes, hydrolytic enzymes, ABC transporters, cadherins, and heat shock proteins. The number of genes in these pathways was fewer than found in flies, such as *D. melanogaster*, and slightly lower than that found in the bumble bees *B. terrestris* and *B. impatiens*, the honey bee *A. mellifera*, and the solitary bee *M. rotundata*. However, a transcriptome may underestimate gene diversity, as compared to studies based on a genome. We also found that, in general, low levels of detoxification and stress related genes are expressed in pupae, adult males and larvae than in adult females. Workers and queens express high levels of P450s and glycosidases.

## Methods

### Source of *B. huntii*

Eight different stages of *B. huntii* were used in this analysis: eggs, early (2nd and 3rd) instar larvae, late (4th) instar larvae, pupae, adult workers, adult males, a diapausing queen, and an egglaying queen. All stages were collected from a nest cultured in the lab at the USDA-ARS Pollinating Insect Research Unit in Logan, UT, except for the diapausing queen, which was a sister of the egglaying queen and had been held in cold storage at 4°C for three months prior to collection for sequencing. The bees were reared according to Strange [66] and were started from queens that were raised and mated in the laboratory. The colony was fed on a diet of pollen collected from honey bee colonies and a 1:1:2 glucose:fructose:sucrose syrup solution. The eggs, larvae and pupae were removed from the colony and killed directly by immersion in RNA*later* solution (Life Technologies, NY, USA), whereas the adult bees were first killed by immersion in liquid nitrogen and were then placed in vials of RNA*later* solution. All bee tissues were submerged in approximately 5 volumes of RNA*later* solution and kept at 4°C overnight to permeate the cells for stabilizing the RNA, the samples were then stored (about one month) at -80°C until processed.

### Preparation of RNA

For RNA isolation, about 1000 eggs, 50 2nd-3rd instar larvae, 20 4th instar larvae, 20 pupae, two adult males, two adult workers, one egglaying queen and one diapausing queen were removed from RNA*later* and washed twice with nuclease free water and then transferred to a mortar and ground in liquid nitrogen to a fine powder. The total RNA was extracted by resuspending the ground powder into 20 ml extraction buffer [100 mM NaCl, 2% SDS, 50 mM Tris–HCl (pH 9.0), 10 mM EDTA (pH 8.0)] and 20 ml phenol-chloroform-isoamyl alcohol (IAA) (49.5:49.5:1, by volume) in a 50 ml centrifuge tube. The solution was mixed and then centrifuged at 8,000 rpm for 20 min at 4°C. The aqueous phase was removed and placed in a clean centrifuge tube and an equal volume of phenol-chloroform-IAA (49.5:49.5:1) was added. The mixture was shaken and then centrifuged at 8,000 rpm for 20 min at 4°C. This organic extraction was repeated two more times. The RNA was precipitated with a 1/10 volume of 3 M sodium acetate (pH 5.2) and 2.5 volumes of 95% ethanol. The RNA pellet was washed with 70% ethanol, dried for 5 min, and resuspended in 400 μl RNase-free water containing 1% diethylpyrocarbonate (DEPC). We repeated total RNA extraction once for each treatment.

Poly(A) + mRNAs were purified with an oligo(dT)-cellulose column through the binding, washing and elution steps. First, 1 ml of total RNA solution (1 mg of RNA for each sample, except ~200 μg for eggs, this was half of the RNA from each extraction) was heated at 65°C for 5 min, then cooled on ice for 5 min, and 200 μl sample buffer [3 M NaCl, 10 mM Tris (pH 7.5), 1 mM EDTA] was added. For the binding step, 8.8 ml of binding solution [0.5 M NaCl, 10 mM Tris (pH 7.5), 0.5% SDS, 0.1 mM EDTA] was added to 1.2 ml RNA sample, agitated for 30 min and then briefly centrifuged to remove the supernatant; all steps were repeated twice more. For the washing step, 10 ml of high salt buffer [0.5 M NaCl, 10 mM Tris (pH 7.5), 1 mM EDTA] were added to the oligo(dT)-cellulose, which was then mixed by rotating 2 min, followed by a brief centrifugation to remove the supernatant. The oligo(dT) was then suspended in 10 ml of high salt buffer and transferred to a 20 ml-column (Bio-Rad, Hercules, CA, USA), washed with the high salt buffer twice, then washed another time with a low salt buffer [0.5 M NaCl, 10 mM Tris (pH 7.5), 1 mM EDTA]. Pre-warmed (65°C) elution buffer [10 mM Tris (pH 7.5), 1 mM EDTA] (3 ml) was added to the top of the oligo(dT)-cellulose for a third time, the suspension was collected, and mRNA was precipitated by adding 50 μl of glycogen solution (20 mg/ml), 1/10 volume of 3 M NaAc (pH 5.2), 7.5 ml of 100% chilled ethanol, and stored overnight at -20°C before being centrifuged. The mRNA pellet was washed with 70% ethanol and dried for 10 min, and then dissolved in 80 μl of RNase-free water (1% DEPC). In addition, the mRNA samples from eggs, larvae, pupae and adult males were amplified using the MessageAmp III RNA amplification kit (Ambion, Austin, TX, USA), creating a sample that was cRNA.

### cDNA library preparation for 454 sequencing

For each sample, a cDNA library was prepared with mRNA or cRNA using a cDNA rapid library preparation kit (Roche, Branford, CT, USA) according to the manufacturer’s instructions, with minor changes. Briefly stated, 18 μl of mRNA or cRNA (around 500 ng) were fragmented using fragmentation solution [0.1 M ZnCl_2_, 0.1 M Tris–HCl (pH 7.0)], followed by vortex mixing and a brief centrifugation, and then heated at 70°C for 30 s. The reaction was stopped by chilling on ice and adding 2 μl of 0.5 M EDTA (pH 8.0) and 28 μl of 10 mM Tris HCl (pH 7.5). The mRNA fragments were purified using 80 μl of RNA Clean reagent, containing SPRI beads (Beckman Coulter, Beverly, MA, USA), and 19 μl of 10 mM Tris HCl (pH 7.5). The beads were removed with centrifugation, and the supernatant containing the RNA was added to a new 200 μl tube.

The first-strand cDNA was synthesized by adding 8 μl 5× RT-buffer AMV, 4 μl 0.1 M DTT, 4 μl 10 mM dNTP, 1 μl protector RNase inhibitor (25 U/μl), 2 μl AMV RT (25 U/μl) to the clean, fragmented RNA, gently mixing, then incubating at 25°C for 10 min, followed by 42°C for 60 min. The second strand cDNA was synthesized by mixing in 30 μl 5× second strand synthesis buffer, 1.5 μl 10 mM dNTPs, 6.5 μl 2^nd^ strand enzyme and 72 μl double distilled water before incubating at 16°C for 2 h, then adding 20 μl T4 DNA polymerase, incubating at 16°C for 5 min, and finally adding 17 μl of 0.2 M EDTA (pH 8.0) to stop the reaction. The double stranded cDNA was purified using AMPure beads (Beckman Coulter, Beverly, MA, USA), and the cDNA was then dissolved in 16 μl of 10 mM Tris–HCl (pH 7.5). The cDNA was further purified using gel purification to isolate fragments of 500–800 bp.

To repair fragment ends, 9 μl of end repair mix (2.5 μl RL (Rapid library) 10× buffer, 2.5 μl RL ATP, 1.0 μl RL dNTP, 1.0 μl RL T4 polymerase, 1.0 μl RL PNK and 1.0 μl RL Taq polymerase), from a cDNA RL preparation kit (Roche, Branford, CT, USA), were added to the cDNA, incubated at 25°C for 20 min, 72°C for 20 min, and then held at 4°C. The adaptor ligation was completed by adding 1 μl of RL adaptor and 1 μl of RL ligase to the reaction tube and incubating at 25°C for 10 min. The small fragments (less than 100 bp) were removed using AMPure beads, and the supernatant contained the cDNA library. The cDNA libraries were then amplified by running emulsion-PCR and sequence analysis performed on a Roche GS-FLX system at the Center for Integrated BioSystems (CIB), Utah State University, Logan, Utah (http://biosystems.usu.edu/).

### Sequence assembly, annotation and detoxification gene identification

The 454 sequence outputs were aligned and assembled *de novo* using CLC Genomics Workbench. The contigs and singletons obtained from *de novo* assemblies were BLAST-searched against the GenBank database at the National Center for Biotechnology Information (NCBI) (http://www.ncbi.nlm.nih.gov/BLAST/) in the iNquiry Bioinformatics Portal. Those similarities maintaining E-values of less than or equal to 0.001 (more than 93% of matches were based on E ≤ e^-05^) were treated as significant matches and were selected as the annotation of *B. huntii* unigenes. The detoxification genes were identified by comparison with detoxification genes found in *A. mellifera*, *D. melanogaster* and other organisms.

### The quality measurement of RNA, cDNA and sequences assembly

The quality and integrity of total RNA, mRNA and cDNA is very important for obtaining high quality transcriptome sequences. The concentration of total RNA was measured using a NanoDrop 2000 Spectrophotometer (Thermo Scientific, Inc. Wilmington, DE, USA), and the quality and integrity of total RNA was examined by electrophoresis. We only used total RNA samples with an A_260_/A_280_ ratio of 2.0 to 2.2 and two typical rRNA bands.

The mRNA concentrations were measured after the first purification from total RNA, and then again after fragmentation for cDNA preparation, using a spectrophotometer (Turner BioSystems, Inc. Sunnyvale, CA USA) with ribogreen RNA reagent (Invitrogen, Foster, CA, USA). The quality and integrity of the mRNA was examined using an Agilent 2100 Bioanalyzer with RNA 6000 Pico kit (Agilent Technologies, Santa Clara, CA, USA). All samples used were of high quality and integrity, as determined by mRNA fluorescence figure with typical shape of broad peak and without two ribosomal RNA contamination peaks. The quality of the fragmented mRNA were determined by running 1 μl of the fragmented mRNA and 1 μl of non-fragmented mRNA on an RNA 6000 Pico Chip on the Agilent 2100 Bioanalyzer. All fragmented samples showed lengths of around 800 bp.

The quality of the cDNA library was determined by the Center for Integrated BioSystems (CIB), Utah State University using a high sensitivity DNA assay on an Agilent Bioanalyzer. All samples displayed a broad shape of peak from 600 bp to 1200 bp with a relatively higher peak at approximately 800 bp. The total read count for the 454 sequence after assembly was 837,010. The average read length was 425 bp with a total read length of 355,789,178 bp.

### Gene diversity and expression levels for detoxification and stress related genes

The diversities of detoxification genes were determined by identifying the number of genes in the particular enzyme group using a BLAST search against the GenBank database at NCBI. Assembled contigs from *B. huntii* that differed from each other in sequence, but matched the same gene in GenBank were considered to be different regions of the same gene if the contigs were each shorter than half the sequence length of the GenBank gene, otherwise they were considered to be different genes.

The expression levels of individual detoxification genes were estimated using RNA-seq [67,68] as follows:

$$\begin{array}{ll} \;\mathrm{Expression}\;\mathrm{level}= & \left(\mathrm{Total}\;\mathrm{reads}\;\mathrm{of}\;\mathrm{the}\;\mathrm{gene}\right)\\ & /\left(\mathrm{Total}\;\mathrm{mapped}\;\mathrm{reads}\;\mathrm{in}\;\mathrm{millions}\;\mathrm{for}\;\mathrm{the}\;\mathrm{sample}\right)\\ & \left(\times \mathrm{gene}\;\mathrm{length}\;\mathrm{in}\;\mathrm{Kb}\right) \end{array}$$

The cumulative expression level for a group of genes was calculated as follows:

$$\begin{array}{ll} \;\mathrm{Expression}\;\mathrm{level}= & \left(\mathrm{Total}\;\mathrm{reads}\;\mathrm{within}\;\mathrm{the}\;\mathrm{group}\;\mathrm{of}\;\mathrm{genes}\right)\\ & /\left(\mathrm{Total}\;\mathrm{mapped}\;\mathrm{reads}\;\mathrm{in}\;\mathrm{millions}\;\mathrm{for}\;\mathrm{the}\;\mathrm{sample}\right)\\ & \left(\times \mathrm{average}\;\mathrm{length}\;\mathrm{of}\;\mathrm{the}\;\mathrm{group}\;\mathrm{of}\;\mathrm{genes}\;\mathrm{in}\;\mathrm{Kb}\right) \end{array}$$

### Availability of supporting data

The sequence data for *B. huntii* cited in this article can be found in the NCBI Sequence Read Archive (SRA), in study #SRP016919, accessions SRX206116 through SRX206124 in. http://sra.dnanexus.com/studies/SRP016919/experiments. The genomic data for *A. mellifera, B. impatiens, B. terrestris, D. melanogaster,* and *M. rotundata* are available at http://www.ncbi.nlm.nih.gov/genome.

## Competing interests

The authors have no competing interests to declare.

## Authors’ contributions

All authors were involved in planning, data interpretation, and writing the manuscript. In addition, JX isolated RNA, constructed the cDNA library, and performed the bioinformatics analysis including assembly of sequences, annotation of unigenes (contigs and singletons set), identification of detoxification genes; JS provided bumble bee samples from insect cultures; and RJ conceived the idea and provided conceptual oversight. All authors have read and approved the final manuscript.

## Supplementary Material

Additional file 1: Table S1 Detoxification and stress response related genes identified in *Bombus huntii*. Click here for file
