# Supplementary material for: Detoxification and stress response genes expressed in a western North American bumble bee, Bombus huntii (Hymenoptera: Apidae)
Source: BMC Genomics. 2013 Dec 12;14:874. doi: 10.1186/1471-2164-14-874 (PMC3878831; doi:10.1186/1471-2164-14-874)
Supplement: Additional file 1: Table S1 — Detoxification and stress response related genes identified in Bombus huntii. [file 1471-2164-14-874-S1.docx]

| **Table S1. Detoxification and stress response related genes from other species identified in *Bombus huntii*.** | | | | | |
| --- | --- | --- | --- | --- | --- |
| **Contig Name** | **Gene ID** | **Seq. Length(nt)** | **Gene Description** | **Species Previously Identified From** | **E-value** |
|  | |  |  |  |  |
| **1. Oxidative-Reductive Enzymes** | |  |  |  |  |
|  |  |  |  |  |  |
| **Catalases** |  |  |  |  |  |
| Contig5780 | gi\|25990773\|gb\|AAN76688.1\| | 1613 | Catalase | *A. mellifera ligustica* | 0.00E+00 |
| G9NSZ3I07H0JYG | gi\|25990773\|gb\|AAN76688.1\| | 417 | Catalase | *A. mellifera ligustica* | 1.00E-14 |
| **Cytochrome P450s** |  |  |  |  |  |
| Contig6528 | gi\|11527279\|gb\|AAG36958.1\| | 1899 | Cytochrome P450 | *Aedes aegypti* | 3.00E-70 |
| Contig23461 | gi\|11527279\|gb\|AAG36958.1\| | 1268 | Cytochrome P450 | *A. aegypti* | 1.00E-52 |
| Contig9334 | gi\|9739177\|gb\|AAF97938.1\| | 1429 | Cytochrome P450 CYP6N3v3 | *A. albopictus* | 3.00E-92 |
| Contig19961 | gi\|9739177\|gb\|AAF97938.1\| | 1468 | Cytochrome P450 CYP6N3v3 | *A. albopictus* | 2.00E-85 |
| Contig19801 | gi\|3249041\|gb\|AAC69184.1\| | 1012 | Corpora allata cytochrome P450 | *Diploptera punctata* | 7.00E-57 |
| Contig8616 | gi\|21914549\|gb\|AAL86019.1\| | 2397 | Cytochrome P450 315a1 | *D. melanogaster* | 2.00E-81 |
| Contig24226 | gi\|14794408\|gb\|AAK73350.1\| | 272 | Cytochrome P450 CYP9J1 | *A. aegypti* | 2.00E-10 |
| Contig226 | gi\|29027552\|gb\|AAO62002.1\| | 2255 | Cytochrome P450 CYPm3r9 | *Anopheles gambiae* | 1.00E-103 |
| Contig6401 | gi\|29027552\|gb\|AAO62002.1\| | 1669 | Cytochrome P450 CYPm3r9 | *A. gambiae* | 6.00E-98 |
| Contig8766 | gi\|29027552\|gb\|AAO62002.1\| | 1989 | Cytochrome P450 CYPm3r9 | *A. gambiae* | 1.00E-82 |
| Contig18547 | gi\|29027552\|gb\|AAO62002.1\| | 2107 | Cytochrome P450 CYPm3r9 | *A. gambiae* | 1.00E-105 |
| Contig19155 | gi\|29027552\|gb\|AAO62002.1\| | 1141 | Cytochrome P450 CYPm3r9 | *A. gambiae* | 5.00E-84 |
| Contig23964 | gi\|14268816\|gb\|AAK57913.1\| | 859 | Cytochrome P450 CYP6J1 | *Blattella germanica* | 2.00E-20 |
| Contig5718 | gi\|14268818\|gb\|AAK57914.1\| | 2979 | Cytochrome P450 CYP6J1 | *B. germanica* | 5.00E-98 |
| Contig9138 | gi\|14268818\|gb\|AAK57914.1\| | 3080 | Cytochrome P450 CYP6J1 | *B. germanica* | 1.00E-126 |
| Contig9968 | gi\|14268818\|gb\|AAK57914.1\| | 1162 | Cytochrome P450 CYP6J1 | *B. germanica* | 2.00E-96 |
| Contig9863 | gi\|14268818\|gb\|AAK57914.1\| | 1300 | Cytochrome P450 CYP6J1 | *B. germanica* | 2.00E-75 |
| Contig13446 | gi\|14268818\|gb\|AAK57914.1\| | 704 | Cytochrome P450 CYP6J1 | *B. germanica* | 8.00E-69 |
| Contig446 | gi\|14582223\|gb\|AAK69410.1\| | 886 | Cytochrome P450 | *B. germanica* | 6.00E-17 |
| Contig5960 | gi\|14582223\|gb\|AAK69410.1\| | 2712 | Cytochrome P450 | *B. germanica* | 6.00E-27 |
| Contig6323 | gi\|14582223\|gb\|AAK69410.1\| | 2612 | Cytochrome P450 | *B. germanica* | 1.00E-127 |
| Contig7805 | gi\|14582223\|gb\|AAK69410.1\| | 1227 | Cytochrome P450 | *B. germanica* | 2.00E-91 |
| Contig7781 | gi\|14582223\|gb\|AAK69410.1\| | 1604 | Cytochrome P450 | *B. germanica* | 6.00E-73 |
| Contig9180 | gi\|14582223\|gb\|AAK69410.1\| | 1681 | Cytochrome P450 | *B. germanica* | 1.00E-108 |
| Contig12587 | gi\|14582223\|gb\|AAK69410.1\| | 686 | Cytochrome P450 | *B. germanica* | 1.00E-38 |
| Contig8652 | gi\|24643092\|ref\|NP_728191.1\| | 1140 | Cytochrome P450-18a1 | *D. melanogaster* | 1.00E-80 |
| Contig9081 | gi\|24643092\|ref\|NP_728191.1\| | 1430 | Cytochrome P450-18a1 | *D. melanogaster* | 6.00E-71 |
| Contig4775 | gi\|17864130\|ref\|NP_524598.1\| | 467 | Cytochrome P450-4c3 | *D. melanogaster* | 5.00E-13 |
| Contig7465 | gi\|33320668\|gb\|AAQ05972.1\| | 696 | Cytochrome P450 CYP314a1 | *D. melanogaster* | 1.00E-17 |
| Contig1854 | gi\|7271937\|gb\|AAF44698.1\| | 465 | Cytochrome P450 CYP6A7 | *Musca domestica* | 6.00E-20 |
| Contig23525 | gi\|14582384\|gb\|AAK69480.1\| | 660 | Cytochrome P450 | *Papilio canadensis* | 9.00E-10 |
| **Contig Name** | **Gene ID** | **Seq. Length(nt)** | **Gene Description** | **Species Previously Identified From** | **E-value** |
| Contig6756 | gi\|585549\|sp\|Q07994\| | 2447 | NADPH-cytochrome P450 reductaseCPR | *M.domestica* | 0.00E+00 |
| HAOP50302B1ZHJ | gi\|3242443\|dbj\|BAA28946.1\| | 525 | Cytochrome P450 | *Culex pipiens quinquefasciatus* | 2.00E-25 |
| HAOP50302B9I8E | gi\|33150238\|gb\|AAP97090.1\| | 520 | Cytochrome P450 CYP4AB1 | *Solenopsis invicta* | 1.00E-07 |
| G9NSZ3I08JQV0A | gi\|11527279\|gb\|AAG36958.1\| | 460 | Cytochrome P450 | *A. aegypti* | 2.00E-15 |
| HAOP50302CMFI8 | gi\|21165972\|gb\|AAM34435.1\| | 512 | Cytochrome P450 | *A. gambiae* | 1.00E-10 |
| G9NSZ3I08JAZNY | gi\|6851332\|gb\|AAF29511.1\| | 280 | Cytochrome P450 | *Trichogramma cacoeciae* | 8.00E-24 |
| HAOP50302B9MWE | gi\|4583521\|gb\|AAD25107.1\| | 479 | Cytochrome P450 CYP6B10 | *Heliothis virescens* | 3.00E-07 |
| HAOP50303DJB43 | gi\|24643092\|ref\|NP_728191.1\| | 487 | Cytochrome P450-18a1 | *D. melanogaster* | 3.00E-11 |
| HAOP50302CBLOL | gi\|22085153\|gb\|AAM90318.1\| | 313 | Cytochrome P450 6B27 | *Helicoverpa zea* | 3.00E-20 |
| HAOP50302CG4BU | gi\|494997\|gb\|AAA82161.1\| | 362 | Cytochrome P450 | *M. domestica* | 2.00E-04 |
| HAOP50303DE3GL | gi\|1706086\|sp\|P50859\| | 495 | Cytochrome P450 51 CYPLI | *Canis gallicus* | 2.00E-39 |
| HAOP50302CI4HG | gi\|3913321\|sp\|O61387\| | 523 | Cytochrome P450 6B7 (CYPVIB7) | *H. armigera* | 6.00E-06 |
| HAOP50302BSBH0 | gi\|11386694\|sp\|Q9V4U7\| | 492 | Cytochrome P450 6a14 (CYPVIA14) | *D. melanogaster* | 6.00E-16 |
| **Dehalogenases** |  |  |  |  |  |
| Contig15887 | gi\|31322698\|gb\|AAP22072.1\| | 701 | Iodotyrosine dehalogenase protein | *H. sapiens* | 7.00E-60 |
| HAOP50302CN4P6 | gi\|24643016\|ref\|NP_728162.1\| | 528 | Haloacid Dehalogenase-like Hydrolases | *D. melanogaster* | 3.00E-13 |
| G9NSZ3I08JH557 | gi\|28571108\|ref\|NP_572257.2\| | 445 | Haloacid dehalogenase-like hydrolases | *D. melanogaster* | 4.00E-10 |
| **Dehydrogenases, alcohol** | |  |  |  |  |
| Contig6180 | gi\|18858257\|ref\|NP_571924.1\| | 1347 | Alcohol dehydrogenase 5 | *Danio rerio* | 1.00E-167 |
| HAOP50304EQZ47 | gi\|18858257\|ref\|NP_571924.1\| | 291 | Alcohol dehydrogenase 5 | *D. rerio* | 3.00E-19 |
| Contig6713 | gi\|40362719\|gb\|AAR84629.1\| | 1228 | Alcohol dehydrogenase | *Gryllotalpa orientalis* | 3.00E-50 |
| Contig7664 | gi\|40362719\|gb\|AAR84629.1\| | 1069 | Alcohol dehydrogenase | *G. orientalis* | 6.00E-57 |
| Contig10510 | gi\|40362719\|gb\|AAR84629.1\| | 1162 | Alcohol dehydrogenase | *G. orientalis* | 1.00E-57 |
| Contig13418 | gi\|40362719\|gb\|AAR84629.1\| | 751 | Alcohol dehydrogenase | *G. orientalis* | 6.00E-40 |
| Contig16055 | gi\|40362719\|gb\|AAR84629.1\| | 1183 | Alcohol dehydrogenase | *G. orientalis* | 1.00E-36 |
| Contig18047 | gi\|30424876\|ref\|NP_780445.1\| | 403 | Fe-containing alcohol dehydrogenase I | *Mus musculus* | 2.00E-19 |
| Contig18278 | gi\|1168346\|sp\|P41747\| | 1273 | Alcohol dehydrogenase I | *Homo sapiens* | 1.00E-124 |
| **Dehydrogenases, aldehyde** | |  |  |  |  |
| Contig7555 | gi\|21914366\|gb\|AAM81354.1\| | 2151 | Aldehyde dehydrogenase | *Steinernema feltiae* | 1.00E-158 |
| Contig7573 | gi\|24586250\|ref\|NP_724560.1\| | 1569 | Aldehyde dehydrogenase type III | *Drosophila melanogaster* | 1.00E-127 |
| Contig10108 | gi\|2494068\|sp\|Q25417\| | 637 | Aldehyde dehydrogenase | *Leishmania tarentolae* | 2.00E-34 |
| HAOP50307IUG2T | gi\|118493\|sp\|P27463\| | 559 | Aldehyde dehydrogenase | *Gallus gallus* | 7.00E-23 |
| G9NSZ3I07IET7H | gi\|17540198\|ref\|NP_502054.1\| | 471 | Aldehyde deHydrogenase alh-3 | *Caenorhabditis elegans* | 1.00E-07 |
| **Dehydrogenases, others** | |  |  |  |  |
| Contig6997 | gi\|24653753\|ref\|NP_611006.1\| | 2010 | FAD/FMN-containing dehydrogenases | *D. melanogaster* | 0.00E+00 |
| Contig23171 | gi\|24660351\|ref\|NP_648149.1\| | 1229 | Acyl-CoA dehydrogenases | *D. melanogaster* | 1.00E-155 |
| HAOP50303DHXCM | gi\|24645648\|ref\|NP_649989.1\| | 498 | Glycine decarboxylase p protein | *D. melanogaster* | 3.00E-64 |
| Contig19169 | gi\|20130025\|ref\|NP_611063.1\| | 1968 | Glycerol-3-phosphate dehydrogenase | *D. melanogaster* | 0.00E+00 |
| **Contig Name** | **Gene ID** | **Seq. Length(nt)** | **Gene Description** | **Species Previously Identified From** | **E-value** |
| Contig6129 | gi\|20130025\|ref\|NP_611063.1\| | 681 | Glycerol-3-phosphate dehydrogenase | *D. melanogaster* | 8.00E-10 |
| Contig11229 | gi\|13528960\|gb\|AAH05270.1\| | 685 | NADH dehydrogenase ubiquinone | *H. sapiens* | 2.00E-43 |
| Contig23541 | gi\|27733925\|ref\|NP_775715.1\| | 1627 | NADH dehydrogenase subunit 4 | *Melipona bicolor* | 5.00E-85 |
| Contig21724 | gi\|17137106\|ref\|NP_477101.1\| | 372 | Succinate dehydrogenase B | *D. melanogaster* | 4.00E-17 |
| Contig15443 | gi\|2282473\|dbj\|BAA21640.1\|1\| | 1196 | Xanthine dehydrogenase | *B. mori* | 3.00E-62 |
| G9NSZ3I07H6MMR | gi\|23478053\|gb\|EAA15243.1\| | 320 | 2-oxoglutarate dehydrogenase, E2 | *Plasmodium yoelii yoelii* | 6.00E-07 |
| Contig10551 | gi\|23308751\|ref\|NP_689953.1\| | 1169 | 3-hydroxyisobutyrate dehydrogenase | *H. sapiens* | 3.00E-91 |
| HAOP50302CE9YC | gi\|2674062\|gb\|AAB88664.1\| | 485 | 3-phosphoglycerate dehydrogenase | *H. sapiens* | 4.00E-21 |
| HAOP50304EAM85 | gi\|29789074\|ref\|NP_058662.1\| | 525 | 3-phosphoglycerate dehydrogenase | *M. musculus* | 7.00E-11 |
| HAOP50303DPORH | gi\|37528837\|gb\|AAQ92344.1\| | 499 | 6-phosphogluconate dehydrogenase | *L. mexicana* | 7.00E-84 |
| Contig46 | gi\|27065480\|pdb\|1LWD\| | 483 | Isocitrate Dehydrogenase | *Sus scrofa* | 6.00E-60 |
| HAOP50302CIM5P | gi\|6435804\|pdb\|3HDH\| | 486 | L-3-Hydroxyacyl Coa Dehydrogenase | *S. scrofa* | 3.00E-52 |
| HAOP50307IWA7E | gi\|17537937\|ref\|NP_495670.1\| | 443 | Branched acid dehydrogenase | *C. elegans* | 2.00E-37 |
| Contig4856 | gi\|24651589\|ref\|NP_651849.1\| | 642 | 2-oxoglutarate dehydrogenase E1 | *D. melanogaster* | 3.00E-15 |
| Contig12673 | gi\|24640492\|ref\|NP_572436.1\| | 497 | Short chain dehydrogenase | *D. melanogaster* | 2.00E-26 |
| Contig5866 | gi\|21357673\|ref\|NP_651578.1\| | 1636 | Short chain dehydrogenase | *D. melanogaster* | 1.00E-145 |
| HAOP50302CGXHM | gi\|24648872\|ref\|NP_651000.1\| | 377 | Isocitrate dehydrogenase | *D. melanogaster* | 2.00E-25 |
| Contig11827 | gi\|19922066\|ref\|NP_610724.1\| | 752 | Short chain dehydrogenase | *D. melanogaster* | 4.00E-44 |
| Contig14532 | gi\|19922066\|ref\|NP_610724.1\| | 551 | Short chain dehydrogenase | *D. melanogaster* | 2.00E-30 |
| Contig940 | gi\|24585512\|ref\|NP_724293.1\| | 1176 | Lactate dehydrogenase | *D. melanogaster* | 1.00E-79 |
| G9NSZ3I07IPE7R | gi\|15077032\|gb\|AAK83037.1\| | 417 | Cytosolic malate dehydrogenase | *T. brucei* | 9.00E-43 |
| HAOP50302B7ED7 | gi\|32189717\|ref\|NP_859447.1\| | 481 | Delta-1-pyroline-5-carboxylate dehydrogenase | *L. major* | 2.00E-76 |
| G9NSZ3I07IQDUC | gi\|1854569\|emb\|CAA72131.1\| | 502 | dihydrolipoamide dehydrogenase | *T. cruzi* | 1.00E-49 |
| Contig17245 | gi\|13272167\|gb\|AAK15809.1\| | 473 | dihydrolipoamide dehydrogenase-like protein | *L. major* | 5.00E-28 |
| Contig3039 | gi\|24645105\|ref\|NP_599138.2\| | 651 | Dihydroorotate dehydrogenase | *D. melanogaster* | 4.00E-57 |
| Contig14453 | gi\|13591940\|ref\|NP_112289.1\| | 1147 | Dihydropyrimidine dehydrogenase | *R. norvegicus* | 1.00E-111 |
| HAOP50307IGQPI | gi\|6014973\|sp\|O08749\| | 660 | Dihydrolipoamide dehydrogenase | *M. musculus* | 5.00E-45 |
| Contig11855 | gi\|2498311\|sp\|Q28943\| | 861 | Dihydropyrimidine dehydrogenase | *S. scrofa* | 1.00E-112 |
| Contig22856 | gi\|26393926\|sp\|O96423\| | 1755 | Glyceraldehyde 3-phosphate dehydrogenase | *C. fasciculata* | 0.00E+00 |
| G9NSZ3I07H1HBG | gi\|28572086\|ref\|NP_524470.3\| | 458 | Glutamate dehydrogenase | *D. melanogaster* | 1.00E-22 |
| Contig1083 | gi\|22450121\|emb\|CAD33827.1\| | 589 | Glyceraldehyde-3-phosphate dehydrogenase | *Plutella xylostella* | 4.00E-27 |
| Contig18517 | gi\|22450121\|emb\|CAD33827.1\| | 1991 | Glyceraldehyde-3-phosphate dehydrogenase | *P. xylostella* | 1.00E-148 |
| HAOP50302BVWZ1 | gi\|31981769\|ref\|NP_034404.2\| | 420 | Glycerol-3-phosphate dehydrogenase 2 | *M. musculus* | 7.00E-19 |
| Contig22784 | gi\|7431252\|pir\|\|JE0234 phosp | 1544 | Hogluconate dehydrogenase | *Ascidia sydneiensis samea* | 0.00E+00 |
| HAOP50302CNVCB | gi\|18700024\|ref\|NP_570954.1\| | 524 | Isocitrate dehydrogenase 3 | *M. musculus* | 1.00E-31 |
| Contig5422 | gi\|5199137\|gb\|AAD40732.1\| | 617 | Lactate dehydrogenase C | *Cricetinae gen. sp.* | 3.00E-52 |
| HAOP50302B0F34 | gi\|387422\|gb\|AAA39509.1\| | 498 | Malate dehydrogenase | *M. musculus* | 1.00E-34 |
| Contig21857 | gi\|5852144\|emb\|CAB55506.1\| | 902 | Malate dehydrogenase | *L. major* | 2.00E-86 |
| **Contig Name** | **Gene ID** | **Seq. Length(nt)** | **Gene Description** | **Species Previously Identified From** | **E-value** |
| HAOP50307IMMBD | gi\|417322\|sp\|Q04448\| | 377 | Bifunctional methylenetetrahydrofolate dehydrogenase | *D. melanogaster* | 4.00E-32 |
| Contig2886 | gi\|28461257\|ref\|NP_787009.1\| | 392 | NADH dehydrogenase ubiquinone | *Bos taurus* | 6.00E-21 |
| HAOP50302B4NEE | gi\|27807355\|ref\|NP_777245.1\| | 444 | NADH dehydrogenase ubiquinone | *B. taurus* | 3.00E-26 |
| HAOP50307IN23L | gi\|27808107\|dbj\|BAC55507.1\| | 402 | NADH dehydrogenase 49 kDa subunit | *Anthoceros formosae* | 7.00E-56 |
| Contig7522 | gi\|4558454\|gb\|AAD22608.1\| | 1100 | NADH dehydrogenase subunit 2 | *A. cerana* | 1.00E-07 |
| Contig19672 | gi\|1786031\|gb\|AAB41179.1\| | 668 | NADH dehydrogenase subunit 2 | *A. mellifera* | 4.00E-04 |
| Contig2915 | gi\|27733923\|ref\|NP_775713.1\| | 608 | NADH dehydrogenase subunit 3 | *M. bicolor* | 1.00E-07 |
| Contig10369 | gi\|27733924\|ref\|NP_775714.1\| | 1334 | NADH dehydrogenase subunit 5 | *M. bicolor* | 3.00E-85 |
| HAOP50304EKAWT | gi\|27733924\|ref\|NP_775714.1\| | 437 | NADH dehydrogenase subunit 5 | *M. bicolor* | 6.00E-16 |
| HAOP50301A8RMC | gi\|453388\|gb\|AAA73418.1\| | 301 | NADH dehydrogenase subunit 7 | *Strigomonas culicis* | 1.00E-27 |
| Contig16119 | gi\|5834938\|ref\|NP_008094.1\| | 1669 | NADH dehydrogenase subunit 1 | *A. mellifera ligustica* | 3.00E-18 |
| Contig20176 | gi\|5834934\|ref\|NP_008090.1\| | 825 | NADH dehydrogenase subunit 4 | *A. mellifera ligustica* | 4.00E-13 |
| Contig20971 | gi\|5834938\|ref\|NP_008094.1\| | 275 | NADH dehydrogenase subunit I | *A. mellifera ligustica* | 1.00E-11 |
| Contig4942 | gi\|1709439\|sp\|P50136\| | 437 | 2-oxoisovalerate dehydrogenase a... | *M. musculus* | 1.00E-46 |
| HAOP50302BS90Y | gi\|3183112\|sp\|P91622\| | 476 | Pyruvate dehydrogenase | *D. melanogaster* | 1.00E-30 |
| HAOP50303DBFQL | gi\|6572421\|emb\|CAB63132.1\| | 458 | Glycerol-3-phosphate dehydrogenase | *L. major* | 4.00E-16 |
| G9NSZ3I07H5JME | gi\|22415742\|gb\|AAM95239.1\| | 435 | NADH dehydrogenase | *T. brucei* | 2.00E-17 |
| Contig11287 | gi\|2507279\|sp\|Q02127\| | 1103 | Dihydroorotate dehydrogenase | *H. sapiens* | 2.00E-40 |
| G9NSZ3I08JC7CS | gi\|4204872\|gb\|AAD11551.1\| | 427 | Pyruvate dehydrogenase E1 alpha subunit | *T. cruzi* | 1.00E-38 |
| Contig22897 | gi\|542577\|pir\|\| | 2661 | Gglutamate dehydrogenase | *D. melanogaster* | 0.00E+00 |
| HAOP50304D54AY | gi\|2146908\|pir\|\| | 346 | NADH2dehydrogenase ubiquinone | *Apis sp.* | 3.00E-05 |
| HAOP50303CYIHJ | gi\|20149748\|ref\|NP_619606.1\| | 512 | Sarcosine dehydrogenase | *M. musculus* | 6.00E-36 |
| HAOP50306HIRVQ | gi\|30353825\|gb\|AAH52123.1\| | 526 | Hydroxyprostaglandin dehydrogenase | *D. rerio* | 6.00E-10 |
| HAOP50302CH0FC | gi\|27769229\|gb\|AAH42315.1\| | 468 | Inosine 5'-phosphate dehydrogenase 2 | *Xenopus laevis* | 6.00E-30 |
| Contig9308 | gi\|3064138\|gb\|AAC14552.1\| | 978 | Sn-glycerol-3-phosphate dehydrogenase | *A. mellifera* | 1.00E-135 |
| Contig12874 | gi\|3064138\|gb\|AAC14552.1\| | 732 | Sn-glycerol-3-phosphate dehydrogenase | *A. mellifera* | 4.00E-27 |
| Contig2837 | gi\|3064138\|gb\|AAC14552.1\| | 312 | Sn-glycerol-3-phosphate dehydrogenase | *A. mellifera* | 2.00E-39 |
| G9NSZ3I08JXML4 | gi\|17737897\|ref\|NP_524311.1\| | 424 | Sorbitol dehydrogenase-2 | *D. melanogaster* | 3.00E-19 |
| Contig10909 | gi\|5931575\|dbj\|BAA84681.1\| | 456 | Succinate dehydrogenase | *T. cruzi* | 2.00E-72 |
| Contig8345 | gi\|18426858\|ref\|NP_569112.1\| | 1095 | Succinate dehydrogenase complex | *R. norvegicus* | 1.00E-95 |
| Contig3667 | gi\|38635910\|emb\|CAA34962.2\| | 509 | Xanthine dehydrogenase | *Calliphora vicina* | 2.00E-36 |
| G9NSZ3I07H0JB4 | gi\|2493964\|sp\|P91711\| | 479 | Xanthine dehydrogenase | *D. subobscura* | 3.00E-30 |
| **Hydroxylases** |  |  |  |  |  |
| Contig14576 | gi\|24654024\|ref\|NP_725525.1\| | 575 | Aspartyl beta-hydroxylase | *D. melanogaster* | 1.00E-16 |
| Contig15510 | gi\|28571133\|ref\|NP_511077.3\| | 504 | Tyramine beta hydroxylase | *D. melanogaster* | 7.00E-47 |
| HAOP50305F0UAI | gi\|17505701\|ref\|NP_492678.1\| | 394 | Fatty acid hydroxylase 36.3 kD | *C.elegans* | 2.00E-08 |
| HAOP50307IOHXB | gi\|21703694\|gb\|AAA49054.2\| | 291 | Cognin/prolyl-4-hydroxylase | *G.gallus* | 7.00E-18 |
| G9NSZ3I07ITDUO | gi\|33304620\|gb\|AAQ02690.1\| | 311 | Aromatic amino acid hydroxylase | *L. major* | 7.00E-04 |
| **Contig Name** | **Gene ID** | **Seq. Length(nt)** | **Gene Description** | **Species Previously Identified From** | **E-value** |
| **Oxidases** |  |  |  |  |  |
| Contig8595 | gi\|29373123\|gb\|AAO72539.1\| | 1026 | Prophenoloxidase | *A. mellifera* | 5.00E-63 |
| Contig6609 | gi\|10697178\|emb\|CAC12696.1\| | 2035 | Prophenoloxidase activating factor | *Tenebrio molitor* | 1.00E-111 |
| Contig5682 | gi\|25989211\|gb\|AAL31707.1\| | 1874 | Prophenoloxidase activating factor 3 | *Bombyx mori* | 3.00E-79 |
| Contig11403 | gi\|1351438\|sp\|P47990\| | 566 | Xanthine dehydrogenase/oxidase I | *G.gallus* | 9.00E-60 |
| Contig23197 | gi\|34881620\|ref\|XP_229131.2\| | 2566 | Hydroquinone NADH oxidase | *Rattus norvegicus* | 3.00E-93 |
| G9NSZ3I08JYVML | gi\|6649587\|gb\|AAF21467.1\| | 423 | Proline oxidase 2 | *M. musculus* | 3.00E-18 |
| G9NSZ3I08JI9N6 | gi\|6448461\|dbj\|BAA86908.1\| | 432 | Lucose oxidase | *A. mellifera* | 5.00E-10 |
| G9NSZ3I07IKQFY | gi\|2133374\|pir\|\|S63687 cytoc | 439 | Hrome-c oxidase precursor | *C. fasciculata* | 2.00E-16 |
| Contig14771 | gi\|21355333\|ref\|NP_648590.1\| | 660 | Galactose oxidase, central domain | *D. melanogaster* | 1.00E-06 |
| Contig2497 | gi\|24582674\|ref\|NP_609180.2\| | 525 | Galactose oxidase, central domain | *D. melanogaster* | 6.00E-17 |
| Contig16887 | gi\|24585081\|ref\|NP_609923.2\| | 459 | hydroxyglutarate oxidase; Provisional | *D. melanogaster* | 9.00E-36 |
| HAOP50302B1OK7 | gi\|24647597\|ref\|NP_650594.1\| | 484 | Galactose oxidase, central domain; | *D. melanogaster* | 2.00E-71 |
| Contig6611 | gi\|417561\|sp\|Q04499\| | 2617 | Proline oxidase, mitochondrial precursor | *D. melanogaster* | 0.00E+00 |
| **Oxidoreductases (quinone reductases, steroid dehydrogenases)** | | |  |  |  |
| HAOP50303DQRI8 | gi\|21355313\|ref\|NP_649811.1\| | 532 | Flavin containing amine oxidoreductase | *D. melanogaster* | 3.00E-42 |
| Contig2674 | gi\|24580572\|ref\|NP_722612.1\| | 505 | NADH:ubiquinone oxidoreductase | *D. melanogaster* | 1.00E-07 |
| Contig10968 | gi\|24662306\|ref\|NP_648409.1\| | 531 | NADH ubiquinone oxidoreductase 13 kDa B | *D. melanogaster* | 2.00E-29 |
| HAOP50307IAUQ4 | gi\|20130091\|ref\|NP_611263.1\| | 497 | FAD dependent oxidoreductase | *D. melanogaster* | 4.00E-32 |
| Contig2689 | gi\|20129243\|ref\|NP_608909.1\| | 471 | NADH:ubiquinone oxidoreductase 13 kDa A | *D. melanogaster* | 6.00E-33 |
| Contig6757 | gi\|17864306\|ref\|NP_524719.1\| | 1001 | NADH:ubiquinone reductase 23kD | *D. melanogaster* | 3.00E-79 |
| HAOP50302CEJB9 | gi\|1172800\|sp\|P42865\| | 496 | Quinine oxidoreductase | *L. amazonensis* | 1.00E-63 |
| Contig3006 | gi\|24653484\|ref\|NP_610914.1\| | 591 | NADPH:quinone reductase and related Zn-dependent oxidoreductases | *D. melanogaster* | 2.00E-55 |
| Contig12101 | gi\|24581339\|ref\|NP_608746.1\| | 775 | NADPH:quinone reductase and related Zn-dependent oxidoreductases | *D. melanogaster* | 6.00E-49 |
| HAOP50303C5Q1M | gi\|8393516\|ref\|NP_057006.1\| | 495 | NADP dependent steroid dehydrogenase-like | *H. sapiens* | 3.00E-04 |
| Contig9116 | gi\|24640442\|ref\|NP_572420.1\| | 1456 | Ketoreductase/ oxidoreductase | *D. melanogaster* | 3.00E-62 |
| **Oxygenases (Mono-, di-, others)** | |  |  |  |  |
| Contig8785 | gi\|24414608\|gb\|AAL40890.1\| | 1251 | Kynurenine 3-monooxygenase | *A. stephensi* | 9.00E-95 |
| Contig9493 | gi\|7682317\|gb\|AAF67216.1\| | 603 | Peptidylglycine alpha-amidating monooxygenase | *Aplysia californica* | 1.00E-16 |
| Contig1709 | gi\|4504381\|ref\|NP_000178.1\| | 721 | H.gentisate 1,2-dioxygenase | *H. sapiens* | 8.00E-67 |
| Contig1643 | gi\|16555097\|gb\|AAL15464.1\| | 585 | Tryptophan oxygenase | *Tribolium castaneum* | 7.00E-32 |
| Contig5905 | gi\|24645709\|ref\|NP_650005.1\| | 1594 | Oxygenase superfamily | *D. melanogaster* | 8.00E-89 |
| Contig6150 | gi\|28572124\|ref\|NP_788714.1\| | 1821 | Non-haem dioxygenase | *D. melanogaster* | 5.00E-63 |
| HAOP50304ER8UL | gi\|28572124\|ref\|NP_788714.1\| | 358 | Non-haem dioxygenase | *D. melanogaster* | 9.00E-06 |
| Contig7362 | gi\|3342705\|gb\|AAC27663.1\| | 1635 | Tryptophan oxygenase | *A. gambiae* | 3.00E-96 |
| Contig10303 | gi\|37595426\|gb\|AAQ94599.1\| | 1105 | Dimethylanaline monooxygenase-like | *D. rerio* | 1.00E-52 |
| Contig6050 | gi\|37595426\|gb\|AAQ94599.1\| | 1701 | Dimethylanaline monooxygenase-like | *D. rerio* | 7.00E-86 |
| **Contig Name** | **Gene ID** | **Seq. Length(nt)** | **Gene Description** | **Species Previously Identified From** | **E-value** |
| G9NSZ3I07IRS2A | gi\|38048309\|gb\|AAR10057.1\| | 388 | Inositol oxygenase activity | *D. yakuba* | 2.00E-10 |
| G9NSZ3I07IOU5L | gi\|38048309\|gb\|AAR10057.1\| | 385 | Inositol oxygenase activity | *D. yakuba* | 2.00E-10 |
| HAOP50303DG1BW | gi\|38048309\|gb\|AAR10057.1\| | 504 | Inositol oxygenase activity | *D. yakuba* | 2.00E-10 |
| G9NSZ3I07INWGH | gi\|38048309\|gb\|AAR10057.1\| | 338 | Inositol oxygenase activity | *D. yakuba* | 8.00E-05 |
| **Peroxidases** |  |  |  |  |  |
| Contig9476 | gi\|17225115\|gb\|AAL37254.1\| | 1001 | 2-Cys thioredoxin peroxidase | *A. aegypti* | 5.00E-90 |
| Contig12785 | gi\|17225115\|gb\|AAL37254.1\| | 1165 | 2-Cys thioredoxin peroxidase | *A. aegypti* | 6.00E-56 |
| Contig6572 | gi\|33089112\|gb\|AAP93585.1\| | 925 | Thioredoxin perxidase | *A. mellifera ligustica* | 6.00E-85 |
| Contig18756 | gi\|33089112\|gb\|AAP93585.1\| | 782 | Thioredoxin perxidase | *A. mellifera ligustica* | 1.00E-57 |
| Contig7383 | gi\|33089110\|gb\|AAP93584.1\| | 1169 | Thioredoxin peroxidase | *A. mellifera ligustica* | 1.00E-118 |
| Contig1172 | gi\|3851500\|gb\|AAC72300.1\| | 471 | Tryparedoxin peroxidase | *Crithidia fasciculata* | 1.00E-34 |
| G9NSZ3I07H2LOQ | gi\|3851500\|gb\|AAC72300.1\| | 446 | Tryparedoxin peroxidase | *C. fasciculata* | 3.00E-76 |
| HAOP50302BWSOK | gi\|3851500\|gb\|AAC72300.1\| | 470 | Tryparedoxin peroxidase | *C. fasciculata* | 9.00E-61 |
| G9NSZ3I07IAMFS | gi\|19171156\|emb\|CAC85915.1\| | 405 | Glutathione peroxidase | *Trypanosoma cruzi* | 9.00E-12 |
| HAOP50303C1IXE | gi\|19171154\|emb\|CAC85914.1\| | 419 | Glutathione peroxidase | *T. cruzi* | 2.00E-14 |
| HAOP50307H5DDV | gi\|3913793\|sp\|O23968\| | 501 | Phospholipid hydroperoxide glutathione peroxidase | *H. armigera* | 6.00E-06 |
| Contig17698 | gi\|28572975\|ref\|NP_788691.1\| | 525 | Peroxiredoxin PRX family | *D. melanogaster* | 2.00E-16 |
| Contig23583 | gi\|28572975\|ref\|NP_788691.1\| | 980 | Peroxiredoxin PRX family | *D. melanogaster* | 1.00E-22 |
| Contig8146 | gi\|14041706\|emb\|CAC38779.1\| | 989 | Glutathione peroxidase | *Suberites domuncula* | 7.00E-71 |
| HAOP50303CZLDV | gi\|28193436\|emb\|CAC83347.1\| | 480 | Glutathione peroxidase-like protein | *T. brucei* | 3.00E-07 |
| HAOP50302B6S0M | gi\|15428288\|gb\|AAK97814.1\| | 604 | Glutathione peroxidase | *Ixodes scapularis* | 2.00E-26 |
| **Superoxide dismutases** | |  |  |  |  |
| Contig5919 | gi\|33089104\|gb\|AAP93581.1\| | 1335 | CuZn superoxide dismutase | *A. mellifera ligustica* | 4.00E-73 |
| Contig7872 | gi\|33089106\|gb\|AAP93582.1\| | 2824 | Mn superoxide dismutase | *A. mellifera ligustica* | 1.00E-113 |
| **Thioredoxins/glutaredoxins** | |  |  |  |  |
| Contig13008 | gi\|23598166\|gb\|AAN34969.1\| | 505 | Thioredoxin 1 | *Onchocerca volvulus* | 1.00E-08 |
| HAOP50302CC9JT | gi\|33089108\|gb\|AAP93583.1\| | 509 | Thioredoxin reductase | *A. mellifera* | 3.00E-29 |
| Contig23373 | gi\|24644513\|ref\|NP_731045.1\| | 2219 | Glutaredoxin GRX family | *D. melanogaster* | 2.00E-64 |
| Contig9630 | gi\|24660041\|ref\|NP_729248.1\| | 605 | Glutaredoxin GRX family | *D. melanogaster* | 5.00E-26 |
|  |  |  |  |  |  |
| **2. Conjugating enzymes** | |  |  |  |  |
|  |  |  |  |  |  |
| **Acetyltransferases** |  |  |  |  |  |
| HAOP50303C5T1N | gi\|24640423\|ref\|NP_572414.1\| | 186 | Acetyl-Coenzyme A acetyltransferase | *D. melanogaster* | 2.00E-15 |
| Contig20748 | gi\|28571773\|ref\|NP_477004.3\| | 386 | Choline acetyltransferase | *D. melanogaster* | 6.00E-28 |
| Contig22976 | gi\|34863356\|ref\|XP_343390.1\| | 1105 | Dihydrolipoamide acetyltransferase | *R. norvegicus* | 8.00E-42 |
| Contig18601 | gi\|34915939\|dbj\|BAC87874.1\| | 1149 | Arylalkylamine N-acetyltransferase | *Periplaneta americana* | 4.00E-45 |
| HAOP50303DL5TQ | gi\|34915939\|dbj\|BAC87874.1\| | 478 | Arylalkylamine N-acetyltransferase | *P.americana* | 2.00E-04 |
| Contig11230 | gi\|24655563\|ref\|NP_611406.1\| | 662 | N-acetyltransferase activity | *D. melanogaster* | 2.00E-04 |
| Contig2932 | gi\|21358519\|ref\|NP_651719.1\| | 812 | N-Acyltransferase superfamily | *D. melanogaster* | 3.00E-41 |
| **Contig Name** | **Gene ID** | **Seq. Length(nt)** | **Gene Description** | **Species Previously Identified From** | **E-value** |
| Contig6426 | gi\|20805283\|gb\|AAM28646.1\| | 2150 | Dihydrolipoamide acetyltransferase precursor | *X. laevis* | 1.00E-119 |
| HAOP50302CAAZ9 | gi\|30842827\|ref\|NP_851595.1\| | 511 | Histone acetyltransferase | *R. norvegicus* | 3.00E-39 |
|  |  |  |  |  |  |
| Contig13513 | gi\|13399322\|ref\|NP_078938.1\| | 1338 | N-acetyltransferase-like | *H. sapiens* | 1.00E-119 |
| Contig16101 | gi\|21312790\|ref\|NP_080646.1\| | 1960 | Histone acetyltransferase 1 | *M. musculus* | 2.00E-77 |
| Contig7879 | gi\|6912512\|ref\|NP_036462.1\| | 1904 | Histone acetyltransferase | *H. sapiens* | 6.00E-40 |
| G9NSZ3I08JVPX6 | gi\|8745054\|emb\|CAB95308.1\| | 464 | Dihydrolipoamide acetyltransferase | *L. major* | 6.00E-40 |
| **Acyltransferases** |  |  |  |  |  |
| Contig13229 | gi\|12804931\|gb\|AAH01918.1\| | 883 | Acetyl-coenzyme A acyltransferase 2 | *H. sapiens* | 2.00E-39 |
| Contig16591 | gi\|12964602\|dbj\|BAB32667.1\| | 557 | Alpha-keto acid dihydrolipoyl acyltransferase | *G. gallus* | 2.00E-30 |
| Contig17399 | gi\|24643305\|ref\|NP_608323.1\| | 607 | Acyltransferase family | *D. melanogaster* | 2.00E-10 |
| Contig12364 | gi\|24641684\|ref\|NP_727670.1\| | 907 | Acyltransferase family | *D. melanogaster* | 9.00E-96 |
| Contig22834 | gi\|19920632\|ref\|NP_608748.1\| | 2982 | Acyl transferase domain | *D. melanogaster* | 0.00E+00 |
| Contig7003 | gi\|31581534\|ref\|NP_060116.2\| | 1201 | tRNA isopentenylpyrophosphate transferase | *H. sapiens* | 3.00E-76 |
| G9NSZ3I07HZPQ8 | gi\|27229019\|ref\|NP_080149.2\| | 380 | tRNA isopentenyltransferase 1 | *M. musculus* | 1.00E-23 |
| **Aminotransferases (transaminase)** | |  |  |  |  |
| Contig21015 | gi\|15425868\|gb\|AAK97625.1\| | 662 | Kynurenine aminotransferase | *A. aegypti* | 5.00E-11 |
| Contig14018 | gi\|19684189\|gb\|AAH26050.1\| | 759 | Ornithine aminotransferase-like 1 | *H. sapiens* | 1.00E-06 |
| Contig5674 | gi\|5360961\|emb\|CAB46365.1\| | 893 | Glucosamine--fructose-6-phosphate aminotransferase | *D. melanogaster* | 1.00E-91 |
| Contig14883 | gi\|6754408\|ref\|NP_035964.1\| | 312 | Kynurenine aminotransferase II | *M. musculus* | 5.00E-15 |
| Contig1813 | gi\|34305305\|gb\|AAQ63487.1\| | 781 | 5-aminoimidazole-4-carboxamideribonucleotide formyltransferase/ | *Leptinotarsa decemlineata* | 1.00E-103 |
| Contig7132 | gi\|17864236\|ref\|NP_524671.1\| | 2505 | Glutamine:fructose-6-phosphate aminotransferase 1 | *D. melanogaster* | 0.00E+00 |
| Contig18634 | gi\|1174432\|sp\|P41689\| | 1747 | Serine--pyruvate aminotransferase | *Felis catus* | 2.00E-96 |
| HAOP50303DSDWR | gi\|12274933\|emb\|CAC22253.1\| | 545 | Alanine:glyoxylate aminotransferase 2 | *H. sapiens* | 1.00E-26 |
| HAOP50302CLPYG | gi\|131612\|sp\|P21872\| | 533 | Glycinamide ribonucleotide synthetase) | G. gallus | 7.00E-25 |
| Contig24621 | gi\|24662918\|ref\|NP_648509.1\| | 316 | Aspartate aminotransferase superfamily | *D. melanogaster* | 4.00E-18 |
| **CoA transferases** |  |  |  |  |  |
| HAOP50303DBS6P | gi\|24655794\|ref\|NP_728699.1\| | 509 | 3-ketoacid CoA transferase | *D. melanogaster* | 2.00E-44 |
| **Formyltransferases** |  |  |  |  |  |
| Contig5935 | gi\|24585660\|ref\|NP_610107.1\| | 2994 | Methionyl-tRNA formyltransferase | *D. melanogaster* | 0.00E+00 |
| HAOP50304ET582 | gi\|24585660\|ref\|NP_610107.1\| | 449 | Methionyl-tRNA formyltransferase | *D. melanogaster* | 2.00E-27 |
| **Glutathione S-transferases** | |  |  |  |  |
| Contig9127 | gi\|32330663\|gb\|AAP79878.1\| | 1254 | Glutathione S-transferase | *S. invicta* | 1.00E-60 |
| Contig22489 | gi\|1346214\|sp\|P42860\| | 1368 | Glutathione S-transferase class-theta | *Lucilia cuprina* | 4.00E-60 |
| Contig21913 | gi\|6225491\|sp\|O18598\| | 419 | Glutathione S-transferase class-sigma | *B. germanica* | 3.00E-24 |
| Contig12465 | gi\|32330663\|gb\|AAP79878.1\| | 727 | Glutathione S-transferase | *S. invicta* | 2.00E-47 |
| HAOP50305FO48F | gi\|32330663\|gb\|AAP79878.1\| | 504 | Glutathione S-transferase | *S. invicta* | 1.00E-13 |
| **Contig Name** | **Gene ID** | **Seq. Length(nt)** | **Gene Description** | **Species Previously Identified From** | **E-value** |
| HAOP50302BXLGR | gi\|32330663\|gb\|AAP79878.1\| | 526 | Gutathione S-transferase | *S. invicta* | 3.00E-17 |
| HAOP50302B2U6N | gi\|1170109\|sp\|P46436\| | 484 | Glutathione S-transferase class-sigma | *Ascaris suum* | 2.00E-09 |
| G9NSZ3I08JR0ES | gi\|6225491\|sp\|O18598\| | 363 | Glutathione S-transferase class-sigma | *B. germanica* | 6.00E-10 |
| HAOP50303C5UYL | gi\|24655311\|ref\|NP_611382.1\| | 521 | Glutathione S-transferase | *D. melanogaster* | 5.00E-67 |
| Contig9271 | gi\|14517793\|gb\|AAK64362.1\| | 1016 | Glutathione-S-transferase-like protein | *Galleria mellonella* | 4.00E-70 |
| Contig16050 | gi\|14517793\|gb\|AAK64362.1\| | 524 | Glutathione-S-transferase-like protein | *G. mellonella* | 4.00E-20 |
| **Glycosyltransferases (glucosyl-, Galactosyl-,Glucuronosyl-,Fucosyl-, Mannosyl-)** | | | |  |  |
| Contig306 | gi\|24657569\|ref\|NP_611636.1\| | 665 | Glycosyl transferase family 21 | *D. melanogaster* | 5.00E-72 |
| Contig472 | gi\|24657569\|ref\|NP_611636.1\| | 417 | Glycosyl transferase family 21 | *D. melanogaster* | 7.00E-33 |
| HAOP50302CHH4J | gi\|24582021\|ref\|NP_723117.1\| | 476 | Glycosyl-transferase for dystroglycan; | *D. melanogaster* | 6.00E-15 |
| HAOP50306HRF9C | gi\|6572204\|emb\|CAB62946.1\| | 427 | Like-glycosyltransferase | *H. sapiens* | 9.00E-43 |
| Contig1331 | gi\|4758664\|ref\|NP_004728.1\| | 938 | Glycosyltransferase | *H. sapiens* | 1.00E-88 |
| Contig16254 | gi\|24645845\|ref\|NP_524313.2\| | 953 | UDP-glycosyltransferase 35b | *D. melanogaster* | 6.00E-45 |
| G9NSZ3I07IGQMP | gi\|24645845\|ref\|NP_524313.2\| | 482 | UDP-glycosyltransferase 35b | *D. melanogaster* | 2.00E-09 |
| Contig2920 | gi\|24647789\|ref\|NP_650662.1\| | 592 | Gycosyltransferases | *D. melanogaster* | 3.00E-37 |
| Contig4129 | gi\|2499087\|sp\|Q09332\| | 741 | UDP-glucose:glycoprotein glucosyltransferase precursor | *D. melanogaster* | 2.00E-34 |
| Contig5586 | gi\|13129070\|ref\|NP_076984.1\| | 469 | Asparagine-linked glycosylation 8 | *H. sapiens* | 4.00E-23 |
| HAOP50304EEHCH | gi\|3297922\|emb\|CAA67521.1\| | 535 | Ecdysteroid UDP-glucosyltransferase | *Spodoptera litura nucleopolyhedrovirus* | 1.00E-09 |
| HAOP50303DDDRS | gi\|29788162\|emb\|CAD88492.1\| | 469 | UDP:Glc glycoprotein glucosyltransferase | *T. cruzi* | 1.00E-23 |
| Contig2628 | gi\|6005952\|ref\|NP_009186.1\| | 409 | Xylosylprotein beta 1,4-galactosyltransferase 7 | *H. sapiens* | 1.00E-31 |
| G9NSZ3I08JX75B | gi\|18079319\|ref\|NP_542172.1\| | 364 | Beta 1,3-galactosyltransferase polypeptide 6 | *H. sapiens* | 5.00E-22 |
| HAOP50302BW13C | gi\|39930599\|ref\|NP_766470.2\| | 493 | Galactosyltransferase 3 beta 1, 4 | *M. musculus* | 5.00E-25 |
| Contig23004 | gi\|30172162\|emb\|CAD89797.1\| | 1756 | Glucuronyltransferase I | *X. laevis* | 3.00E-74 |
| Contig16387 | gi\|24584982\|ref\|NP_609882.1\| | 302 | UDP-glucosyl transferase | *D. melanogaster* | 2.00E-09 |
| Contig9707 | gi\|14456077\|emb\|CAC41641.1\| | 624 | Alpha 1,3-fucosyltransferase | *D. melanogaster* | 9.00E-59 |
| Contig6364 | gi\|34576296\|emb\|CAE46766.1\| | 1003 | Core3-alpha-L-fucosyltransferase | *Glossina morsitans* | 2.00E-56 |
| Contig13874 | gi\|23489993\|gb\|EAA21870.1\| | 323 | Mannosyltransferase-related | *P. yoelii* | 2.00E-06 |
| **Methyltransferases** |  |  |  |  |  |
| HAOP50302BXQS1 | gi\|6684525\|gb\|AAF23609.1\| | 548 | DNA (cytosine-5)-methyltransferase | *H. sapiens* | 6.00E-79 |
| Contig12368 | gi\|8132067\|gb\|AAF73200.1\| | 474 | DNA-(cytosine-5)-methyltransferase | *Xiphophorus maculatus x X.helleri* | 1.00E-30 |
| G9NSZ3I07IAS22 | gi\|29568124\|gb\|AAO61495.1\| | 475 | Leucine carboxyl methyltransferase | *Takifugu rubripes* | 4.00E-15 |
| Contig17577 | gi\|28497773\|ref\|XP_125730.2\| | 795 | Histone methyltransferase | *M. musculus* | 3.00E-39 |
| G9NSZ3I08JOQ2D | gi\|482365\|pir\|\| | 428 | O-methyltransferase | *B. taurus* | 1.00E-13 |
| HAOP50305GIQCO | gi\|482365\|pir\|\| | 500 | O-methyltransferase | *B. taurus* | 2.00E-15 |
| Contig2407 | gi\|28574557\|ref\|NP_788012.1\| | 540 | Acetylserotonin O-methyltransferase | *D. melanogaster* | 1.00E-26 |
| Contig20441 | gi\|37728049\|gb\|AAO44952.1\| | 555 | Cytosine-5-methyltransferase | *B. taurus* | 3.00E-29 |
| Contig12242 | gi\|4894862\|gb\|AAD32631.1\| | 1455 | Denovo DNA methyltransferase 3 | *D. rerio* | 2.00E-78 |
| Contig8490 | gi\|28572120\|ref\|NP_524452.4\| | 1116 | Diphthamide methyltransferase | *G.gallus* | 2.00E-81 |
| **Contig Name** | **Gene ID** | **Seq. Length(nt)** | **Gene Description** | **Species Previously Identified From** | **E-value** |
| HAOP50304EKMQE | gi\|25143613\|ref\|NP_740808.1\| | 506 | Dot1l Histone Methyltransferase | *C. elegans* | 1.00E-03 |
| Contig15266 | gi\|40217808\|ref\|NP_079033.3\| | 462 | Euchromatic histone methyltransferase 1 | *H. sapiens* | 2.00E-42 |
| HAOP50302B858E | gi\|818905\|gb\|AAA67080.1\| | 562 | Farnesoic acid o-methyltransferase | *Homarus americanus* | 9.00E-10 |
| Contig11096 | gi\|29029589\|ref\|NP_803183.1\| | 732 | rRNA uridine-2'-O-)-methyltransferase | *H. sapiens* | 4.00E-60 |
| HAOP50304ED1AH | gi\|417042\|sp\|P28337\| | 534 | Aminomethyltransferase | *G.gallus* | 7.00E-21 |
| HAOP50302CIKH4 | gi\|13385938\|ref\|NP_080716.1\| | 219 | RNA guanine-7-) methyltransferase | *M. musculus* | 2.00E-09 |
| Contig13293 | gi\|34861169\|ref\|XP_341956.1\| | 577 | DNA cytosine-5)-methyltransferase 3A | *R. norvegicus* | 2.00E-24 |
| HAOP50302CM8TH | gi\|34872789\|ref\|XP_340846.1\| | 416 | RNA methyltransferase | *R. norvegicus* | 2.00E-05 |
| **Phosphotransferases** | |  |  |  |  |
| Contig22907 | gi\|21356313\|ref\|NP_651372.1\| | 1004 | Phosphotransferase enzyme family | *D. melanogaster* | 1.00E-25 |
| Contig8305 | gi\|33328839\|gb\|AAQ09826.1\| | 1475 | Phosphotransferase enzyme family | *D. yakuba* | 5.00E-32 |
| G9NSZ3I07H4AT0 | gi\|32699620\|sp\|Q9NJP9\| | 346 | Glycerol 3-phosphotransferase | *T. brucei* | 5.00E-13 |
| **Sulfotransferases** |  |  |  |  |  |
| HAOP50307IHPKJ | gi\|24645281\|ref\|NP_649870.1\| | 378 | Sulfotransferase domain | *D. melanogaster* | 4.00E-22 |
| Contig22509 | gi\|20301936\|ref\|NP_609043.1\| | 299 | Sulfotransferase domain | *D. melanogaster* | 2.00E-08 |
| **Other transferases (phosphoribosyl-, Nucleotidyl-, Palmitoyl-)** | | | |  |  |
| Contig23667 | gi\|601907\|gb\|AAA57204.1\| | 762 | Adenine phosphoribosyltransferase | *D. melanogaster* | 1.00E-42 |
| G9NSZ3I07IRDHW | gi\|38492453\|pdb\|1MZV\| | 441 | Phosphoribosyltransferase | *L. tarentolae* | 3.00E-07 |
| Contig531 | gi\|33860180\|sp\|O43938\| | 578 | S-adenosylmethionine synthetase | *L. donovani* | 1.00E-97 |
| Contig17152 | gi\|12311845\|emb\|CAC22663.1\| | 493 | tRNA nucleotidyltransferase precursor | *L. major* | 8.00E-29 |
| HAOP50307ICG3T | gi\|21357561\|ref\|NP_647622.1\| | 367 | CTP:phosphocholine cytidylyltransferase 2 | *D. melanogaster* | 2.00E-15 |
| HAOP50302CF7DP | gi\|24665024\|ref\|NP_648837.1\| | 530 | Nucleotidyl transferase superfamily | *D. melanogaster* | 1.00E-40 |
| Contig7293 | gi\|4504035\|ref\|NP_003866.1\| | 1800 | Guanine monophosphate synthetase | *H. sapiens* | 0.00E+00 |
| Contig16806 | gi\|21313396\|ref\|NP_084232.1\| | 861 | Adenosyltransferase | *M. musculus* | 3.00E-24 |
| Contig6179 | gi\|24580597\|ref\|NP_608508.1\| | 1719 | Palmitoyltransferase | *D. melanogaster* | 1.00E-52 |
| Contig24441 | gi\|5821160\|dbj\|BAA83721.1\| | 743 | Serine palmitoyl transferase | *D. melanogaster* | 2.00E-70 |
| HAOP50302CNEKT | gi\|34873871\|ref\|XP_225180.2\| | 301 | Serine C-palmitoyltransferase | *R. norvegicus* | 3.00E-18 |
| Contig23834 | gi\|17738187\|ref\|NP_524494.1\| | 580 | Oligosaccharyl transferase 3 | *D. melanogaster* | 1.00E-71 |
| HAOP50303C3IN4 | gi\|21358211\|ref\|NP_647705.1\| | 477 | Peptide O-xylosyltransferase | *D. melanogaster* | 2.00E-42 |
| G9NSZ3I07H0S4T | gi\|6066425\|emb\|CAB58292.1\| | 457 | Mannose-1-phosphate guanyltransferase | *L. major* | 1.00E-64 |
| G9NSZ3I08JXJ0R | gi\|23491407\|gb\|EAA22948.1\| | 375 | N-acetylglucosamine transferase | *P. yoelii yoelii* | 2.00E-10 |
| HAOP50302BWEPY | gi\|34042922\|gb\|AAQ56700.1\| | 460 | N-acetylgalactosaminyltransferase | *D. melanogaster* | 8.00E-72 |
| Contig14483 | gi\|22538453\|ref\|NP_683721.1\| | 502 | N-acetylglucosaminyl transferase | *H. sapiens* | 6.00E-21 |
| Contig13550 | gi\|22002033\|sp\|O95260\| | 631 | Arginyl-tRNA--protein transferase | *H. sapiens* | 1.00E-19 |
| G9NSZ3I07IEXQV | gi\|7544113\|dbj\|BAA94298.1\| | 391 | Aspartate carbamoyltransferase | *L. mexicana amazonensis* | 7.00E-12 |
| HAOP50302CM7WE | gi\|1703311\|sp\|P52183\| | 527 | Gamma-glutamyltransferase | *Schistocerca americana* | 7.00E-11 |
| Contig18589 | gi\|345423\|pir\|\| | 1036 | Gamma-glutamyltransferase | *Tachypleus tridentatus* | 5.00E-66 |
| Contig18922 | gi\|1703311\|sp\|P52183\| | 2558 | Gamma-glutamyltransferase | *S. americana* | 0.00E+00 |
| **Contig Name** | **Gene ID** | **Seq. Length(nt)** | **Gene Description** | **Species Previously Identified From** | **E-value** |
| **3. Hydrolytic Enzymes** | |  |  |  |  |
|  |  |  |  |  |  |
| **Acid phosphatases** |  |  |  |  |  |
| Contig7398 | gi\|22335687\|dbj\|BAC10547.1\| | 1178 | Acid phosphatase | *D. virilis* | 8.00E-42 |
| Contig10241 | gi\|189619\|gb\|AAA60022.1\| | 862 | Acid phosphatase | *H. sapiens* | 1.00E-20 |
| Contig14093 | gi\|9790059\|ref\|NP_062774.1\| | 496 | Acid phosphatase 6 | *M. musculus* | 2.00E-07 |
| G9NSZ3I08I50V5 | gi\|2058524\|gb\|AAC79513.1\| | 464 | Histidine secretory acid phosphatase | *L. donovani* | 3.00E-58 |
| HAOP50307IEHWY | gi\|17864600\|ref\|NP_524917.1\| | 495 | Acid phosphatase 1 | *D. melanogaster* | 5.00E-10 |
| HAOP50302B7JQQ | gi\|11359724\|pir\|\| | 484 | Secreted acid phosphatase 2 precursor | *L. mexicana* | 4.00E-32 |
| G9NSZ3I07H4X57 | gi\|13518080\|gb\|AAK27378.1\| | 485 | Phosphatidic acid phosphatase-like protein | *L. donovani* | 5.00E-04 |
| **Alkaline phosphatases** | |  |  |  |  |
| Contig5813 | gi\|24651554\|ref\|NP_524601.2\| | 1791 | Alkaline phosphatase 4 | *D. melanogaster* | 1.00E-115 |
| **Amidases** |  |  |  |  |  |
| Contig7265 | gi\|24648433\|ref\|NP_650893.1\| | 1826 | Asp-tRNAAsn/Glu-tRNAGln amidotransferase A | *D. melanogaster* | 3.00E-86 |
| Contig21867 | gi\|28194049\|gb\|AAO33382.1\| | 829 | Dihydropyrimidine amidohydrolase | *D. melanogaster* | 9.00E-92 |
| **Aminopeptidase/other peptidases (Endo-, Di-, Carboxy-)** | | |  |  |  |
| Contig11570 | gi\|584751\|sp\|Q07075\| | 991 | Glutamyl aminopeptidase | *H. sapiens* | 6.00E-68 |
| Contig6181 | gi\|19335622\|gb\|AAL85580.1\| | 3049 | Aminopeptidase N | *A. aegypti* | 1.00E-149 |
| Contig8653 | gi\|3402300\|dbj\|BAA32140.1\| | 1612 | Aminopeptidase N | *B. mori* | 6.00E-63 |
| HAOP50302B81EZ | gi\|27734472\|emb\|CAD20931.1\| | 519 | Aminopeptidase N | *H. sapiens* | 1.00E-08 |
| Contig12812 | gi\|24650973\|ref\|NP_733277.1\| | 1153 | Aminopeptidase N family | *D. melanogaster* | 3.00E-47 |
| G9NSZ3I08I7JMX | gi\|4107172\|emb\|CAA10526.1\| | 242 | Aminopeptidase P | *D. melanogaster* | 2.00E-12 |
| Contig9149 | gi\|27806565\|ref\|NP_776523.1\| | 642 | Leucine aminopeptidase 3 | *B. taurus* | 5.00E-45 |
| Contig6668 | gi\|24655260\|ref\|NP_728616.1\| | 3048 | Puromycin sensitive aminopeptidase | *D. melanogaster* | 1.00E-148 |
| Contig20316 | gi\|24655260\|ref\|NP_728616.1\| | 787 | Puromycin sensitive aminopeptidase | *D. melanogaster* | 1.00E-120 |
| Contig8725 | gi\|18875372\|ref\|NP_573479.1\| | 896 | X-prolyl aminopeptidase aminopeptidase P | *M. musculus* | 8.00E-36 |
| Contig14028 | gi\|38075464\|ref\|XP_110411.2\| | 826 | Aminopeptidase ZK353.6 | *M. musculus* | 4.00E-41 |
| G9NSZ3I07IQ91O | gi\|7706387\|ref\|NP_057218.1\| | 481 | Aminopeptidase | *H. sapiens* | 8.00E-04 |
| Contig6054 | gi\|13359138\|dbj\|BAB33300.1\| | 2814 | Neutral endopeptidase 24.11 | *B. mori* | 0.00E+00 |
| Contig6705 | gi\|13359138\|dbj\|BAB33300.1\| | 900 | Neutral endopeptidase 24.11 | *B. mori* | 1.00E-16 |
| Contig17888 | gi\|13786144\|ref\|NP_112614.1\| | 537 | Prolyl endopeptidase | *R. norvegicus* | 1.00E-62 |
| Contig11045 | gi\|4506185\|ref\|NP_002780.1\| | 1002 | Multicatalytic endopeptidase | *H. sapiens* | 1.00E-109 |
| Contig18977 | gi\|542655\|pir\|\| | 1023 | Proteasome endopeptidase complex | *X. laevis* | 2.00E-95 |
| Contig2675 | gi\|21358201\|ref\|NP_650318.1\| | 479 | Dipeptidase B | *D. melanogaster* | 8.00E-17 |
| HAOP50302BYSZG | gi\|21357079\|ref\|NP_650192.1\| | 393 | Dipeptidase C | *D. melanogaster* | 8.00E-40 |
| Contig6575 | gi\|5713150\|gb\|AAD47827.1\| | 1939 | Carboxypeptidase A | *A. aegypti* | 3.00E-55 |
| Contig22052 | gi\|13751094\|emb\|CAC37108.1\| | 710 | Thermostable carboxypeptidase 3 | *L. major* | 1.00E-55 |
| Contig1184 | gi\|34857839\|ref\|XP_214993.2\| | 846 | Lysosomal Pro-X carboxypeptidase | *R. norvegicus* | 7.00E-86 |
| Contig6543 | gi\|13928880\|ref\|NP_113828.1\| | 1122 | Plasma glutamate carboxypeptidase | *R. norvegicus* | 7.00E-99 |
| **Contig Name** | **Gene ID** | **Seq. Length(nt)** | **Gene Description** | **Species Previously Identified From** | **E-value** |
| Contig6758 | gi\|115881\|sp\|P04069\| | 1551 | Carboxypeptidase B | *Astacus fluviatilis* | 9.00E-85 |
| Contig19222 | gi\|115881\|sp\|P04069\| | 1643 | Carboxypeptidase B | *A. fluviatilis* | 1.00E-70 |
| G9NSZ3I07IP0R2 | gi\|7511718\|pir\|\| | 302 | Carboxypeptidase D | *A. californica* | 3.00E-21 |
| G9NSZ3I07IPCN6 | gi\|34869409\|ref\|XP_221358.2\| | 497 | Carboxypeptidase N | *R. norvegicus* | 4.00E-14 |
| G9NSZ3I08JLYI0 | gi\|35181448\|gb\|AAO74600.1\| | 454 | Serine carboxypeptidase precursor | *T. cruzi* | 3.00E-31 |
| HAOP50301BBYJF | gi\|4877698\|gb\|AAD31418.1\| | 445 | Glutamate carboxypeptidase precursor | *H. sapiens* | 4.00E-34 |
| G9NSZ3I08I9DNL | gi\|6978699\|ref\|NP_036968.1\| | 317 | Carboxypeptidase D | *R. norvegicus* | 1.00E-27 |
| HAOP50301A1DWE | gi\|21356111\|ref\|NP_651314.1\| | 473 | Zn-dependent oligopeptidases | *D. melanogaster* | 8.00E-20 |
| HAOP50302CBAY6 | gi\|13122207\|emb\|CAB89585.2\| | 483 | Dipeptidyl-peptidase III | *L. major* | 3.00E-72 |
| Contig19339 | gi\|13592121\|ref\|NP_112399.1\| | 1372 | Tripeptidylpeptidase II | *R. norvegicus* | 1.00E-35 |
| HAOP50302B5DON | gi\|11065741\|emb\|CAB95256.2\| | 482 | Mitochondrial processing peptidase | *L. major* | 2.00E-30 |
| Contig20067 | gi\|24667786\|ref\|NP_649271.1\| | 409 | Zn-dependent peptidases | *D. melanogaster* | 3.00E-17 |
| Contig12894 | gi\|3183167\|sp\|Q28250\| | 728 | Microsomal signal | *Canis sp.* | 3.00E-42 |
| Contig23577 | gi\|6978513\|ref\|NP_036632.1\| | 1120 | N-acylaminoacyl-peptide hydrolase | *R. norvegicus* | 2.00E-30 |
| **α-amylases** |  |  |  |  |  |
| Contig18577 | gi\|20377081\|gb\|AAM20738.1\| | 2209 | Alpha-amylase | *A. mellifera ligustica* | 0.00E+00 |
| Contig1962 | gi\|28573410\|ref\|NP_788342.1\| | 562 | Alpha amylase | *D. melanogaster* | 9.00E-81 |
| **Carboxylesterases and esterases** | |  |  |  |  |
| Contig22282 | gi\|12003419\|gb\|AAG43568.1\| | 512 | Acetylcholinesterase | *A. mellifera* | 2.00E-27 |
| Contig14831 | gi\|12958609\|gb\|AAK09373.1\| | 549 | Acetylcholinesterase precursor | *Schizaphis graminum* | 6.00E-16 |
| G9NSZ3I07IT3VK | gi\|40363516\|dbj\|BAD06210.1\| | 362 | Acetylcholinesterase | *C. tritaeniorhynchus* | 9.00E-46 |
| Contig6078 | gi\|3153849\|gb\|AAC36245.1\| | 1789 | Carboxylesterase | *Anisopteromalus calandrae* | 1.00E-149 |
| Contig19030 | gi\|3153849\|gb\|AAC36245.1\| | 1585 | Carboxylesterase | *A. calandrae* | 2.00E-68 |
| Contig23116 | gi\|3153849\|gb\|AAC36245.1\| | 1158 | Carboxylesterase | *A. calandrae* | 9.00E-46 |
| HAOP50301A5W5D | gi\|3153849\|gb\|AAC36245.1\| | 533 | Carboxylesterase | *A. calandrae* | 2.00E-26 |
| G9NSZ3I07IFWI4 | gi\|4768933\|gb\|AAD29685.1\| | 391 | Carboxylesterase | *M. domestica* | 9.00E-07 |
| Contig2487 | gi\|24649826\|ref\|NP_651304.1\| | 832 | Esterases and lipases | *D. melanogaster* | 1.00E-81 |
| Contig22326 | gi\|21357257\|ref\|NP_652674.1\| | 746 | Phospholipase/Carboxylesterase | *D. melanogaster* | 7.00E-76 |
| Contig18910 | gi\|24586385\|ref\|NP_610326.1\| | 960 | Esterases and lipases | *D. melanogaster* | 3.00E-76 |
| HAOP50303C50FX | gi\|21357257\|ref\|NP_652674.1\| | 474 | Phospholipase/Carboxylesterase | *D. melanogaster* | 7.00E-26 |
| Contig24115 | gi\|24582257\|ref\|NP_609051.1\| | 550 | Esterases and lipases | *D. melanogaster* | 5.00E-15 |
| Contig6235 | gi\|17646748\|gb\|AAL41023.1\| j | 1697 | Uvenile hormone esterase | *T. molitor* | 2.00E-59 |
| Contig9735 | gi\|29465748\|gb\|AAM14416.1\| | 962 | Integumental esterase | *Antheraea polyphemus* | 8.00E-71 |
| Contig2447 | gi\|34870074\|ref\|XP_341026.1\| | 336 | Neuropathy target esterase | *R. norvegicus* | 3.00E-12 |
| Contig5350 | gi\|544255\|sp\|P35501\| | 792 | Esterase E4 precursor | *Myzus persicae* | 1.00E-49 |
| Contig17445 | gi\|5020383\|gb\|AAD38067.1\| | 1060 | Juvenile hormone esterase binding protein | *Manduca sexta* | 1.00E-09 |
| HAOP50302CIZT5 | gi\|29465748\|gb\|AAM14416.1\| | 538 | Integumental esterase | *A. polyphemus* | 5.00E-07 |
| HAOP50303C5JDZ | gi\|6716737\|gb\|AAF26723.1\| | 405 | Alpha-esterase 2 | *D. buzzatii* | 8.00E-34 |
| HAOP50303CZR9U | gi\|9927565\|gb\|AAG02021.1\| | 437 | Alpha-esterase like protein E4 | *T. castaneum* | 6.00E-24 |
| HAOP50302B07GZ | gi\|33944889\|ref\|XP_340592.1\| | 416 | Esterase | *T. brucei brucei* | 7.00E-11 |
| **Contig Name** | **Gene ID** | **Seq. Length(nt)** | **Gene Description** | **Species Previously Identified From** | **E-value** |
| HAOP50303C8VSC | gi\|544256\|sp\|P35502\| | 518 | Esterase FE4 precursor | *M. persicae* | 4.00E-33 |
| **Cyclohydrolases** |  |  |  |  |  |
| Contig23382 | gi\|1079083\|pir\|\| | 1023 | GTP cyclohydrolase | *D. melanogaster* | 2.00E-57 |
| **Glycosidases (Glucosidases, Glucuronidases)** | |  |  |  |  |
| HAOP50303C1SEA | gi\|18693231\|dbj\|BAA25890.2\| | 504 | Acid alpha glucosidase | *Coturnix japonica* | 5.00E-39 |
| Contig14940 | gi\|3097290\|dbj\|BAA25884.1\| | 792 | Acid alpha glucosidase | *C. japonica* | 5.00E-47 |
| Contig18527 | gi\|20334294\|dbj\|BAB91145.1\| | 1923 | Beta-glucosidase | *Neotermes koshunensis* | 1.00E-133 |
| Contig9095 | gi\|6225644\|sp\|Q17058\|\| | 1938 | Alpha-glucosidase precursor Maltase | *A. mellifera* | 0.00E+00 |
| Contig20564 | gi\|6225644\|sp\|Q17058\|\| | 772 | Alpha-glucosidase precursor Maltase | *A. mellifera* | 6.00E-17 |
| Contig7272 | gi\|21357605\|ref\|NP_652145.1\| | 2549 | Glucosidase II | *D. melanogaster* | 0.00E+00 |
| Contig7043 | gi\|19921464\|ref\|NP_609844.1\| | 2587 | Glucosidase II beta subunit-like | *D. melanogaster* | 1.00E-108 |
| Contig11729 | gi\|24656935\|ref\|NP_726061.1\| | 1092 | Glycogen debranching enzyme | *D. melanogaster* | 1.00E-111 |
| Contig18590 | gi\|28571438\|ref\|NP_788858.1\| | 3955 | Alpha-glucosidase glucoamylase | *D. melanogaster* | 0.00E+00 |
| Contig6035 | gi\|38503165\|sp\|Q9BDT0\| | 1698 | Glucosylceramidase precursor | *Pan troglodytes* | 1.00E-107 |
| Contig17714 | gi\|38503165\|sp\|Q9BDT0\| | 519 | Glucosylceramidase precursor | *P. troglodytes* | 2.00E-17 |
| Contig16169 | gi\|19922582\|ref\|NP_611395.1\| | 2357 | Beta-D-glucuronidase | *D. melanogaster* | 0.00E+00 |
| **Glycosylases** |  |  |  |  |  |
| Contig17637 | gi\|4325213\|gb\|AAD17300.1\| | 402 | Uracil DNA glycosylase | *X. laevis* | 2.00E-30 |
| Contig6594 | gi\|21356805\|ref\|NP_651925.1\| | 2510 | Specific DNA glcosylase | *D. melanogaster* | 2.00E-99 |
| Contig4419 | gi\|28571383\|ref\|NP_788889.1\| | 516 | Glycosyl hydrolases family 15 | *D. melanogaster* | 2.00E-12 |
| **Hydrolases** |  |  |  |  |  |
| HAOP50304EG2SN | gi\|30686425\|ref\|NP_194147.2\| | 500 | Hydrolase, alpha/beta fold family | *A. thaliana* | 7.00E-36 |
| Contig10207 | gi\|6981424\|ref\|NP_037145.1\| | 699 | Prosaposinr | *R. norvegicus* | 3.00E-08 |
| Contig18505 | gi\|418129\|sp\|P32359\| | 3133 | Trehalase precursor | T. molitor | 1E-149 |
| G9NSZ3I08JRF9Q | gi\|27806491\|ref\|NP_776563.1\| | 447 | Poly ADP-ribose) glycohydrolase | *B. taurus* | 4.00E-07 |
| Contig6806 | gi\|24648688\|ref\|NP_650962.1\| | 657 | Isopentenyldiphosphate isomerase | *D. melanogaster* | 2.00E-45 |
| Contig20580 | gi\|28571729\|ref\|NP_650453.3\| | 742 | 3-hydroxyisobutyryl-CoA hydrolase | *D. melanogaster* | 8.00E-51 |
| Contig6439 | gi\|37681823\|gb\|AAQ97789.1\| | 1931 | Hydroxyacyl glutathione hydrolase | *D. rerio* | 2.00E-86 |
| **Nitrilases** |  |  |  |  |  |
| G9NSZ3I07IQLO0 | gi\|21355835\|ref\|NP_649888.1\| | 421 | Nitrilase activity | *D. melanogaster* | 3.00E-13 |
| G9NSZ3I07IS1Q2 | gi\|21355835\|ref\|NP_649888.1\| | 444 | Nitrilase activity | *D. melanogaster* | 1.00E-13 |
| HAOP50301A0K23 | gi\|21355835\|ref\|NP_649888.1\| | 527 | Nitrilase activity | *D. melanogaster* | 3.00E-20 |
| HAOP50301A6YG8 | gi\|21355835\|ref\|NP_649888.1\| | 484 | Nitrilase activity | *D. melanogaster* | 2.00E-10 |
| HAOP50304D5ADQ | gi\|21355835\|ref\|NP_649888.1\| | 517 | Nitrilase activity | *D. melanogaster* | 3.00E-15 |
| HAOP50304EA6QO | gi\|21355835\|ref\|NP_649888.1\| | 475 | Nitrilase activity | *D. melanogaster* | 1.00E-13 |
| HAOP50304EU7K2 | gi\|21355835\|ref\|NP_649888.1\| | 492 | Nitrilase activity | *D. melanogaster* | 3.00E-22 |
| HAOP50305FRMHL | gi\|21355835\|ref\|NP_649888.1\| | 542 | Nitrilase activity | *D. melanogaster* | 9.00E-19 |
| HAOP50305GCDGT | gi\|21355835\|ref\|NP_649888.1\| | 545 | Nitrilase activity | *D. melanogaster* | 5.00E-14 |
| HAOP50305GHW4F | gi\|21355835\|ref\|NP_649888.1\| | 541 | Nitrilase activity | *D. melanogaster* | 3.00E-14 |
| G9NSZ3I08I8LB5 | gi\|21355835\|ref\|NP_649888.1\| | 438 | Nitrilase activity | *D. melanogaster* | 0.001 |
| **Contig Name** | **Gene ID** | **Seq. Length(nt)** | **Gene Description** | **Species Previously Identified From** | **E-value** |
| G9NSZ3I08I9FAF | gi\|21355835\|ref\|NP_649888.1\| | 425 | Nitrilase activity | *D. melanogaster* | 0.001 |
| G9NSZ3I08JP416 | gi\|21355835\|ref\|NP_649888.1\| | 443 | Nitrilase activity | *D. melanogaster* | 0.001 |
| G9NSZ3I08JUZI0 | gi\|21355835\|ref\|NP_649888.1\| | 423 | Nitrilase activity | *D. melanogaster* | 0.001 |
| HAOP50301BE79Z | gi\|21355835\|ref\|NP_649888.1\| | 480 | Nitrilase activity | *D. melanogaster* | 1.00E-14 |
| HAOP50302B9YJF | gi\|21355835\|ref\|NP_649888.1\| | 539 | Nitrilase activity | *D. melanogaster* | 2.00E-16 |
| HAOP50302CB19N | gi\|21355835\|ref\|NP_649888.1\| | 539 | Nitrilase activity | *D. melanogaster* | 2.00E-16 |
| HAOP50302CDM1J | gi\|21355835\|ref\|NP_649888.1\| | 522 | Nitrilase activity | *D. melanogaster* | 3.00E-22 |
| HAOP50304EH1U5 | gi\|21355835\|ref\|NP_649888.1\| | 508 | Nitrilase activity | *D. melanogaster* | 1.00E-10 |
| HAOP50304EPAVQ | gi\|21355835\|ref\|NP_649888.1\| | 534 | Nitrilase activity | *D. melanogaster* | 1.00E-13 |
| HAOP50305F5W23 | gi\|21355835\|ref\|NP_649888.1\| | 527 | Nitrilase activity | *D. melanogaster* | 8.00E-18 |
| HAOP50305GB2CJ | gi\|21355835\|ref\|NP_649888.1\| | 533 | Nitrilase activity | *D. melanogaster* | 3.00E-13 |
| HAOP50306G6HW2 | gi\|21355835\|ref\|NP_649888.1\| | 578 | Nitrilase activity | *D. melanogaster* | 2.00E-17 |
| HAOP50306G85KQ | gi\|21355835\|ref\|NP_649888.1\| | 522 | Nitrilase activity | *D. melanogaster* | 1.00E-14 |
| HAOP50306HEKQP | gi\|21355835\|ref\|NP_649888.1\| | 483 | Nitrilase activity | *D. melanogaster* | 0.00004 |
| **Phosphodiesterases** |  |  |  |  |  |
| Contig1548 | gi\|33151017\|gb\|AAP49573.1\| | 494 | cAMP phosphodiesterase | *T. cruzi* | 4.00E-10 |
| Contig14822 | gi\|24581359\|ref\|NP_523465.2\| | 787 | Tyrosyl-DNA phosophodiesterase 1 | *D. melanogaster* | 2.00E-21 |
| Contig2069 | gi\|15027090\|emb\|CAC44911.1\| | 428 | Possible phosphodiesterase | *L. major* | 1.00E-50 |
| G9NSZ3I07HZ2V8 | gi\|27372879\|dbj\|BAC53765.1\| | 371 | Phosphodiesterase 8B4 | *H. sapiens* | 4.00E-07 |
| HAOP50303DPG5V | gi\|6679249\|ref\|NP_032829.1\| | 510 | Phosphodiesterase 8A | *M. musculus* | 4.00E-34 |
| HAOP50303DSR5E | gi\|1236959\|gb\|AAA97892.1\| | 425 | cAMP-specific phoshodiesterase | *H. sapiens* | 7.00E-27 |
| Contig3850 | gi\|14248761\|gb\|AAK57641.1\| | 593 | cAMP-specific cyclic nucleotide phosphodiesterase | *H. sapiens* | 3.00E-34 |
| HAOP50307IRM4F | gi\|6679245\|ref\|NP_032827.1\| | 408 | Phosphodiesterase 6D | *M. musculus* | 4.00E-15 |
| **Phosphohydrolases** |  |  |  |  |  |
| Contig4679 | gi\|125977\|sp\|P16621\| | 512 | Phosphohydrolase | *D. melanogaster* | 2.00E-15 |
| Contig13826 | gi\|4502125\|ref\|NP_001152.1\| | 662 | P4-tetraphosphate pyrophosphohydrolase | *H. sapiens* | 2.00E-39 |
| HAOP50304EIORQ | gi\|118913\|sp\|P16620\| | 460 | Phosphohydrolase | *D. melanogaster* | 1.00E-17 |
| HAOP50301BC08P | gi\|4758972\|ref\|NP_004568.1\| | 379 | L-3-phosphoserine phosphatase | *H. sapiens* | 7.00E-19 |
| HAOP50303C6TBW | gi\|5870609\|emb\|CAB55619.1\| | 138 | Sucrose-6-phosphate hydrolase | *L. major* | 3.00E-08 |
| **Other esterases (Pectin-, Methyl-)** | |  |  |  |  |
| HAOP50303C2PPA | gi\|27544452\|dbj\|BAC54964.1\| | 434 | Pectinmethylesterase inhibitor | *Actinidia deliciosa* | 2.00E-12 |
| Contig1415 | gi\|30794138\|ref\|NP_082568.1\| | 596 | Protein phosphatase methylesterase 1 | *M. musculus* | 6.00E-44 |
| Contig5584 | gi\|30794138\|ref\|NP_082568.1\| | 460 | Protein phosphatase methylesterase 1 | *M. musculus* | 2.00E-40 |
| HAOP50303DR82Q | gi\|6688852\|emb\|CAB65291.1\| | 484 | Pectin methyl-esterase PEF1 | *M. truncatula* | 1.00E-35 |
| **Other hydrolases (Ubiquitin carboxyl-terminal, Peptidyl-tRNA and AdenosylH.cysteine hydrolases)** | | | |  |  |
| Contig19815 | gi\|19922206\|ref\|NP_610917.1\| | 928 | Ubiquitin C-terminal hydrolase | *D. melanogaster* | 6.00E-37 |
| HAOP50306HM0G8 | gi\|19922206\|ref\|NP_610917.1\| | 475 | Ubiquitin C-terminal hydrolase | *D. melanogaster* | 3.00E-06 |
| Contig20302 | gi\|24647481\|ref\|NP_650561.1\| | 498 | Peptidyl-tRNA hydrolase, type 2 | *D. melanogaster* | 2.00E-25 |
| HAOP50303DGV7R | gi\|24647481\|ref\|NP_650561.1\| | 301 | Peptidyl-tRNA hydrolase, type 2 | *D. melanogaster* | 2.00E-07 |
| Contig926 | gi\|1710837\|sp\|P36889\| | 494 | AdenosylH.cysteinase | *L. donovani* | 1.00E-84 |
| **Contig Name** | **Gene ID** | **Seq. Length(nt)** | **Gene Description** | **Species Previously Identified From** | **E-value** |
| HAOP50307IKEG2 | gi\|30584089\|gb\|AAP36293.1\| | 200 | S-adenosylH.cysteine hydrolase | *Synthetic construct* | 5.00E-04 |
|  |  |  |  |  |  |
| **4. Other enzymes associated with detoxification** | |  |  |  |  |
|  |  |  |  |  |  |
| **Cadherins** |  |  |  |  |  |
| Contig18576 | gi\|517102\|dbj\|BAA05942.1\| | 5243 | DE-cadherin | *D. melanogaster* | 0.00E+00 |
| Contig1297 | gi\|23956250\|ref\|NP_536685.1\| | 749 | Cadherin EGF LAG seven-pass G-type receptor 3 | *M. musculus* | 1.00E-05 |
| Contig8838 | gi\|28893539\|ref\|NP_796353.1\| | 2360 | Beta -catenin-N-cadherin | *M. musculus* | 5.00E-15 |
| HAOP50303DCO8S | gi\|31201207\|ref\|XP_309551.1\| | 506 | Catenin cadherin-associated protein | *A. gambiae* | 8.00E-39 |
| HAOP50304EPA3Q | gi\|7459679\|pir\|\| | 482 | DN-cadherin | *D. melanogaster* | 4.00E-28 |
| Contig8722 | gi\|24665868\|ref\|NP_648973.1\| | 962 | Cadherin tandem repeat domain; | *D. melanogaster* | 3.00E-26 |
| **Heat shock proteins** | |  |  |  |  |
| HAOP50302B3KEX | gi\|37515266\|gb\|AAH00004.2\| | 523 | J-type co-chaperone HSC20 | *H. sapiens* | 7.00E-24 |
| HAOP50304ENRLU | gi\|4507713\|ref\|NP_003306.1\| | 474 | Hsp40 H.log, subfamily C | *H. sapiens* | 2.00E-28 |
| Contig18440 | gi\|32880141\|gb\|AAP88901.1\| | 2214 | Hsp40 H.log, subfamily A | *Synthetic construct* | 1.00E-129 |
| Contig23826 | gi\|40254873\|ref\|NP_054868.2\| | 1248 | HSPC049 protein | *H. sapiens* | 5.00E-36 |
| Contig14647 | gi\|32526896\|ref\|NP_056211.2\| | 991 | HSPC056 protein | *H. sapiens* | 2.00E-92 |
| contig22921 | gi\|31231072\|ref\|XP_318461.1\| | 2607 | 60 kDa heat shock protein | *A. gambiae* | 0.00E+00 |
| Contig8968 | gi\|3023478\|sp\|Q95046\| | 1194 | Chaperonin HSP60, mitochondrial precursor | *T. cruzi* | 1.00E-176 |
| Contig8588 | gi\|37993866\|gb\|AAP57537.3\| | 1324 | Heat shock protein 70 | *Locusta migratoria* | 2.00E-90 |
| Contig8856 | gi\|9864199\|gb\|AAG01344.1\| | 831 | Heat shock protein 70 | *L. braziliensis* | 7.00E-57 |
| Contig1640 | gi\|3386646\|gb\|AAC28558.1\| | 254 | Heat shock protein 70 | *L. braziliensis* | 2.00E-32 |
| HAOP50305F1XU8 | gi\|3004463\|emb\|CAA04673.1\| | 537 | Heat shock protein 70 | *Oreochromis mossambicus* | 1.00E-17 |
| HAOP50305F2452 | gi\|7363334\|gb\|AAF61296.1\| | 457 | Heat shock protein 70 | *Clathrina clatrus* | 6.00E-15 |
| HAOP50305GJMM2 | gi\|33319729\|gb\|AAQ05768.1\| | 479 | Heat shock protein 70 | *Penaeus monodon* | 2.00E-17 |
| Contig18593 | gi\|27260894\|gb\|AAN86047.1\| | 2881 | Heat shock cognate 70 protein | *S. frugiperda* | 0.00E+00 |
| Contig3220 | gi\|23193450\|gb\|AAN14525.1\| | 581 | Heat shock cognate 70 | *Chironomus tentans* | 4.00E-61 |
| Contig15764 | gi\|266312\|sp\|P29845\| | 430 | Heat shock 70 KD protein cognates | *D. melanogaster* | 9.00E-23 |
| Contig23253 | gi\|12585261\|sp\|Q9U639\| | 2846 | Heat shock 70 kDa protein cognate | *M. sexta* | 0.00E+00 |
| HAOP50304ETIZK | gi\|38327039\|ref\|NP_002145.3\| | 417 | Heat shock 70kDa protein 4 isoform a | *H. sapiens* | 5.00E-20 |
| Contig410 | gi\|1078698\|pir\|\| | 307 | DnaK-type molecular chaperone hsp70 | *L. donovani infantum* | 6.00E-42 |
| Contig19390 | gi\|21245112\|ref\|NP_640354.1\| | 558 | Hsp70-interacting protein | *R. norvegicus* | 1.00E-12 |
| contig19047 | gi\|31212601\|ref\|XP_315285.1\| | 1440 | Heat shock 70 kDa protein 4L-like | *A. gambiae* | 1.00E-164 |
| contig9660 | gi\|31212601\|ref\|XP_315285.1\| | 833 | Heat shock 70 kDa protein 4L-like | *A. gambiae* | 3.00E-41 |
| G9NSZ3I07ISWXW | gi\|25553516\|dbj\|BAC24979.1\| | 366 | Mitochondrial HSP70 | *T. congolense* | 2.00E-50 |
| HAOP50305F65L0 | gi\|21427282\|gb\|AAM53166.1\| | 481 | Hsp70 protein | *Alopias vulpinus* | 2.00E-23 |
| HAOP50302CJRIN | gi\|33944507\|ref\|XP_340401.1\| | 471 | Mitochondrial heat shock protein 78 | *T. brucei* | 6.00E-65 |
| Contig17686 | gi\|1066807\|gb\|AAB05638.1\| | 254 | Heat shock protein 82 | *A. albimanus* | 1.00E-40 |
| HAOP50307ID1Q2 | gi\|17647529\|ref\|NP_523899.1\| | 565 | Heat shock protein 83 | *D. melanogaster* | 6.00E-33 |
| **Contig Name** | **Gene ID** | **Seq. Length(nt)** | **Gene Description** | **Species Previously Identified From** | **E-value** |
| Contig6329 | gi\|21542414\|sp\|Q25293\| | 2718 | Heat shock protein 83-1HSP 83 | *L. infantum* | 0.00E+00 |
| Contig18892 | gi\|12005809\|gb\|AAG44630.1\| | 1101 | 90-kDa heat shock protein HSP83 | *S. frugiperda* | 1.00E-141 |
| Contig10217 | gi\|12005809\|gb\|AAG44630.1\| | 799 | 90-kDa heat shock protein HSP83 | *S. frugiperda* | 1.00E-114 |
| Contig5788 | gi\|72221\|pir\|\| | 2409 | HHCH90 heat shock protein 90 | *G. gallus* | 0.00E+00 |
| HAOP50305F49UQ | gi\|20177936\|sp\|Q9GKX8\| | 514 | Heat shock protein HSP 90 | *Equus caballus* | 4.00E-27 |
| Contig8539 | gi\|21542002\|sp\|Q24740\| | 837 | Hsp90 co-chaperone | *D. virilis* | 6.00E-72 |
| Contig10061 | gi\|1946209\|emb\|CAA86116.1\| | 1696 | 100 kDa heat shock protein Hsp100 | *L. major* | 2.00E-96 |
| Contig23146 | gi\|2058336\|emb\|CAB08073.1\| | 1360 | Heat shock protein 100 | *L. donovani* | 0.00E+00 |
| HAOP50304EU98L | gi\|2495362\|sp\|Q94738\| | 460 | Heat shock protein 110 | *S. franciscanus* | 3.00E-22 |
| Contig11300 | gi\|7661790\|ref\|NP_054886.1\| | 916 | HSPC128 protein | *H. sapiens* | 1.00E-10 |
| Contig14498 | gi\|27696459\|gb\|AAH44029.1\| | 524 | Hspc150-prov protein | *X. laevis* | 2.00E-29 |
| G9NSZ3I08JHW1H | gi\|6841574\|gb\|AAF29140.1\| | 378 | HSPC177 | H. sapiens | 1.00E-19 |
| HAOP50303C73N4 | gi\|7661832\|ref\|NP_054907.1\| | 501 | HSPC182 protein | H. sapiens | 2.00E-58 |
| Contig19099 | gi\|7106790\|gb\|AAF36120.1\| | 1062 | HSPC200 | *H. sapiens* | 2.00E-34 |
| Contig4364 | gi\|7106826\|gb\|AAF36138.1\| | 711 | HSPC218 | *H. sapiens* | 2.00E-09 |
| G9NSZ3I08JRKCA | gi\|6841194\|gb\|AAF28950.1\| | 392 | HSPC272 | *H. sapiens* | 3.00E-24 |
| Contig2882 | gi\|27732311\|ref\|XP_215838.1\| | 560 | HSPC274 protein | *R. norvegicus* | 5.00E-26 |
| Contig362 | gi\|28566306\|gb\|AAO43053.1\| | 549 | Heat shock regulated-1 | *H. sapiens* | 1.00E-63 |
| HAOP50303DMLSX | gi\|38082228\|ref\|XP_207062.2\| | 446 | Heat shock protein 1A | *M. musculus* | 8.00E-23 |
| G9NSZ3I08I9EOY | gi\|12311804\|emb\|CAC22622.1\| | 330 | Co-chaperone or heat shock protein | *L. major* | 6.00E-21 |
| Contig8007 | gi\|4503729\|ref\|NP_002005.1\| | 747 | HSP binding immunophilin | *H. sapiens* | 3.00E-63 |
| contig23356 | gi\|24643312\|ref\|NP_728275.1\| | 661 | Heat shock proteins (Hsps) | *D. melanogaster* | 6.00E-44 |
| HAOP50302CFW60 | gi\|24643312\|ref\|NP_728275.1\| | 484 | Heat shock proteins (Hsps) | *D. melanogaster* | 5.00E-36 |
| HAOP50302CK0NY | gi\|24643312\|ref\|NP_728275.1\| | 481 | Heat shock proteins (Hsps) | *D. melanogaster* | 4.00E-32 |
| HAOP50303CZT8X | gi\|24643312\|ref\|NP_728275.1\| | 493 | Heat shock proteins (Hsps) | *D. melanogaster* | 1.00E-36 |
| **Heavy metal associated domain** | |  |  |  |  |
| Contig6980 | gi\|24641427\|ref\|NP_572756.1\| | 4213 | Heavy-metal-associated domain HMA | *D. melanogaster* | 0.00E+00 |
| **Isomerases** |  |  |  |  |  |
| Contig10082 | gi\|20378696\|gb\|AAM20942.1\| | 483 | Triosephosphate isomerase | *L. infantum* | 4.00E-20 |
| Contig10828 | gi\|22090453\|emb\|CAD43178.1\| | 1338 | Triosephosphate isomerase | *T. molitor* | 2.00E-99 |
| HAOP50307ICT5P | gi\|34221743\|emb\|CAE45561.1\| | 688 | Triosephosphate isomerase | *Loboptera decipiens* | 1.00E-27 |
| Contig3585 | gi\|6094494\|sp\|O16140\| | 810 | DNA Topoisomerase II | *B. mori* | 1.00E-120 |
| Contig6910 | gi\|6755851\|ref\|NP_035754.1\| | 1077 | Topoisomerase DNA III beta | *M. musculus* | 1.00E-105 |
| Contig12285 | gi\|28422192\|gb\|AAH46848.1\| | 518 | Topoisomerase DNA III beta | *X. laevis* | 2.00E-60 |
| G9NSZ3I08JIB1K | gi\|1786132\|dbj\|BAA19101.1\| | 455 | DNA topoisomerase I | *G. gallus* | 1.00E-54 |
| HAOP50304EEEBO | gi\|24640096\|ref\|NP_511059.2\| | 472 | Topoisomerase 3beta | *D. melanogaster* | 1.00E-27 |
| HAOP50302CLCO8 | gi\|5902138\|ref\|NP_008958.1\| | 387 | Topoisomerase DNA II binding protein | *H. sapiens* | 3.00E-11 |
| G9NSZ3I08JZ0C3 | gi\|6094494\|sp\|O16140\| | 314 | DNA Topoisomerase II | *B. mori* | 2.00E-37 |
| Contig23940 | gi\|6677767\|ref\|NP_033101.1\| | 745 | Ribose 5-phosphate isomerase A | *M. musculus* | 7.00E-24 |
| Contig18607 | gi\|1730180\|sp\|P52031\| | 2023 | Glucose-6-phosphate isomerase | *D. yakuba* | 0.00E+00 |
| **Contig Name** | **Gene ID** | **Seq. Length(nt)** | **Gene Description** | **Species Previously Identified From** | **E-value** |
| Contig7740 | gi\|17647799\|ref\|NP_524079.1\| | 1089 | Protein disulfide isomerase | *P. falciparum* | 1.00E-104 |
| Contig20162 | gi\|37497116\|ref\|NP_922915.1\| | 457 | Protein disulfide isomerase- | *D. rerio* | 6.00E-34 |
| Contig5939 | gi\|1706258\|sp\|P54985\| | 1656 | Peptidyl-prolyl cis-trans isomerase | *B. germanica* | 5.00E-78 |
| HAOP50303DOK2W | gi\|8388702\|emb\|CAB94114.1\| | 324 | Peptidylprolyl isomerase | *L. major* | 6.00E-10 |
| G9NSZ3I08JIQCO | gi\|11342604\|emb\|CAC17142.1\| | 392 | Phosphomannose isomerase | *L. mexicana* | 2.00E-46 |
| Contig3595 | gi\|1170958\|sp\|Q09734\| | 628 | Peptidyl-prolyl cis-trans isomerase | *T. cruzi* | 8.00E-29 |
| HAOP50304EBB7Q | gi\|2499773\|sp\|Q26486\| | 478 | Peptidyl-prolyl cis-trans isomerase | *S. frugiperda* | 5.00E-19 |
| **Lyases** |  |  |  |  |  |
| Contig8895 | gi\|24639685\|ref\|NP_572164.2\| | 2107 | Class II terpene cyclases | *D. melanogaster* | 2.00E-68 |
| Contig17390 | gi\|2406633\|gb\|AAB70469.1\| | 1061 | Adenylyl cyclase isoform DAC9 | *D. melanogaster* | 1.00E-27 |
| Contig2302 | gi\|8392852\|ref\|NP_058838.1\| | 422 | Adenylyl cyclase 8 | *R. norvegicus* | 8.00E-20 |
| Contig23101 | gi\|3687839\|gb\|AAC62238.1\| | 3476 | NO-insensitive guanylyl cyclase | *M. Sexta* | 1.00E-131 |
| Contig6208 | gi\|12964646\|dbj\|BAB32672.1\| | 2379 | Receptor type guanylyl cyclase | *B. mori* | 1.00E-176 |
| HAOP50302CODRL | gi\|12964646\|dbj\|BAB32672.1\| | 522 | Receptor type guanylyl cyclase | *B. mori* | 3.00E-06 |
| Contig14244 | gi\|3372756\|gb\|AAC61264.1\| | 815 | Soluble guanylyl cyclase beta-1 subunit | *M. sexta* | 4.00E-46 |
| G9NSZ3I08JMISZ | gi\|32394696\|gb\|AAM94353.1\| | 145 | Guanylyl cyclase receptor | *Bactrocera dorsalis* | 1.00E-16 |
| HAOP50302CBKGI | gi\|38015990\|dbj\|BAD00154.1\| | 496 | Membrane guanylyl cyclase2 | *Oryzias curvinotus* | 7.00E-09 |
| HAOP50302B8XMZ | gi\|33413439\|gb\|AAK97794.1\| | 516 | Soluble guanylate cyclase subunit | *A. californica* | 6.00E-52 |
| Contig2000 | gi\|7512218\|pir\|\| | 539 | Guanylate cyclase | *G. gallus* | 9.00E-06 |
| HAOP50302BYSVF | gi\|1362612\|pir\|\| | 534 | Guanylate cyclase | *D. melanogaster* | 3.00E-09 |
| G9NSZ3I07H5XE2 | gi\|27414115\|gb\|AAO12127.1\| | 408 | Guanylate cyclase | *L. donovani* | 6.00E-05 |
| G9NSZ3I08JRRWB | gi\|4557257\|ref\|NP_001106.1\| | 322 | Adenylate cyclase 8 | *H. sapiens* | 6.00E-31 |
| HAOP50305FQY64 | gi\|232149\|sp\|P30676\| | 114 | Adenylate cyclase-inhibiting G alpha protein | *Asterina pectinifera* | 1.00E-06 |
| Contig18968 | gi\|29789122\|ref\|NP_066940.1\| | 1006 | Cryptochrome 2 photolyase-like | *H. sapiens* | 1.00E-136 |
| HAOP50303DLAI0 | gi\|12311881\|emb\|CAC22697.1\| | 375 | Adenylosuccinate lyase | *L. major* | 5.00E-63 |
| HAOP50303DI8D9 | gi\|133504\|sp\|P27864\| | 450 | Recombination repair protein 1 DNA-apurinic or apyrimidinic site; lyase | *D. melanogaster* | 6.00E-11 |
| G9NSZ3I08JHACJ | gi\|3023613\|sp\|Q25263\| | 495 | Receptor-type adenylate cyclase B | *L. donovani* | 2.00E-21 |
| **Lysosomal enzymes** |  |  |  |  |  |
| Contig15982 | gi\|14861380\|gb\|AAK73655.1\| | 680 | Lysosomal alpha-N-acetyl glucosaminidase | *Dromaius novaehollandiae* | 1.00E-65 |
| HAOP50302CNOC3 | gi\|5869912\|emb\|CAB55547.1\| | 475 | Lysosomal acid phosphatase | *L. major* | 1.00E-30 |
| HAOP50302CIXEB | gi\|6754592\|ref\|NP_034878.1\| | 455 | Lysosomal trafficking regulator | *M. musculus* | 8.00E-43 |
| G9NSZ3I08JB27Z | gi\|16758280\|ref\|NP_445970.1\| | 378 | Lysosomal trafficking regulator | *R. norvegicus* | 2.00E-06 |
| Contig8578 | gi\|4757355\|dbj\|BAA77267.1\| | 2112 | Lysosomal trafficking regulator | *B. taurus* | 2.00E-12 |
| Contig6524 | gi\|19921088\|ref\|NP_609408.1\| | 844 | Lysosomal alpha-mannosidase | *D. melanogaster* | 1.00E-102 |
| HAOP50302BVAHK | gi\|24583443\|ref\|NP_723591.1\| | 545 | Lysosomal alpha-mannosidase | *D. melanogaster* | 2.00E-60 |
| **Transporters, ABC** |  |  |  |  |  |
| Contig6959 | gi\|2959643\|gb\|AAC05632.1\| | 2746 | ABC transporter | *H. sapiens* | 2.00E-18 |
| Contig2469 | gi\|18478576\|gb\|AAL73206.1\| | 475 | ABCA1.2 transporter | *L. tropica* | 2.00E-31 |
| Contig21965 | gi\|13182799\|gb\|AAK14943.1\| | 458 | ABCA1 transporter | *T. cruzi* | 3.00E-13 |
| Contig379 | gi\|11493656\|gb\|AAG35594.1\| | 368 | ABC1 transporter | *L. tropica* | 3.00E-35 |
| **Contig Name** | **Gene ID** | **Seq. Length(nt)** | **Gene Description** | **Species Previously Identified From** | **E-value** |
| HAOP50302BW4P7 | gi\|3355757\|emb\|CAA08835.1\| | 531 | ABC transporter protein | *G. gallus* | 4.00E-40 |
| HAOP50305GDV9S | gi\|24638558\|ref\|NP_726547.1\| | 514 | ABC transporter transmembrane region | *D. melanogaster* | 2.00E-43 |
| Contig16106 | gi\|30023643\|gb\|AAO18684.1\| | 8831 | ATP-binding cassette transporter | *M. musculus* | 0.00E+00 |
| Contig6480 | gi\|13517534\|gb\|AAK28835.1\| | 2370 | ATP-binding cassette transporter G1 variant III | *H. sapiens* | 1.00E-118 |
| HAOP50303C7LJ4 | gi\|22218268\|gb\|AAM94613.1\| | 525 | ATP-binding cassette transporter subfamily A | *Strongylocentrotus purpuratus* | 5.00E-25 |
| HAOP50302BXUE4 | gi\|5032101\|ref\|NP_005679.1\| | 522 | ATP-binding cassette, sub-family C | *H. sapiens* | 7.00E-44 |
| Contig12822 | gi\|11342541\|emb\|CAC17140.1\| | 741 | White family ATP-binding cassette transporter | *H. sapiens* | 8.00E-54 |
| **Transporters, others (Dopamine, Glucose, Glutamate, Monocarboxylate, Phosphate)** | | | |  |  |
| Contig3136 | gi\|29468059\|gb\|AAN52844.1\| | 513 | Dopamine transporter | *Trichoplusia ni* | 2.00E-21 |
| HAOP50302CB1D7 | gi\|8571432\|gb\|AAF76882.1\| | 413 | Dopamine transporter | *D. melanogaster* | 1.00E-24 |
| Contig15474 | gi\|34860785\|ref\|XP_345472.1\| | 920 | Glucose transporter 10 | *R. norvegicus* | 2.00E-17 |
| Contig6414 | gi\|17737517\|ref\|NP_523878.1\| | 2584 | Glucose transporter 1 | *D. melanogaster* | 0.00E+00 |
| Contig22875 | gi\|17737517\|ref\|NP_523878.1\| | 1723 | Glucose transporter 1 | *D. melanogaster* | 1.00E-118 |
| Contig17407 | gi\|23821304\|dbj\|BAC20934.1\| | 467 | Glucose transporter type 8 | *G. gallus* | 3.00E-10 |
| Contig275 | gi\|4960026\|gb\|AAD34586.1\| | 521 | Glutamate transporter | *A. mellifera* | 1.00E-57 |
| Contig6446 | gi\|8050559\|gb\|AAF71701.1\| | 2444 | Na+-dependent glutamate transporter | *Diploptera punctata* | 1.00E-141 |
| HAOP50303DHMJV | gi\|8050559\|gb\|AAF71701.1\| | 361 | Na+-dependent glutamate transporter | *D. punctata* | 1.00E-13 |
| Contig8171 | gi\|5453704\|ref\|NP_006398.1\| | 656 | Glutamate transporter | *H. sapiens* | 5.00E-16 |
| HAOP50307H8ALB | gi\|2352298\|gb\|AAB84380.1\| | 488 | Na+-dependent glutamate transporter | *T. ni* | 3.00E-26 |
| Contig2417 | gi\|13122224\|emb\|CAC32260.1\| | 475 | Monocarboxylate transporter protein | *L. major* | 2.00E-19 |
| Contig23977 | gi\|24639306\|ref\|NP_477334.2\| | 874 | Monocarboxylate transporter 1 | *D. melanogaster* | 4.00E-59 |
| HAOP50301A1LZ9 | gi\|24639306\|ref\|NP_477334.2\| | 481 | Monocarboxylate transporter 1 | *D. melanogaster* | 2.00E-49 |
| Contig5938 | gi\|28475243\|emb\|CAD59636.1\| | 1781 | Na+-dependent inorganic phosphate cotransporter | *D. melanogaster* | 1.00E-133 |
